# Common Genetic Variation In Cellular Transport Genes and Epithelial Ovarian Cancer (EOC) Risk

**DOI:** 10.1371/journal.pone.0128106

**Published:** 2015-06-19

**Authors:** Ganna Chornokur, Hui-Yi Lin, Jonathan P. Tyrer, Kate Lawrenson, Joe Dennis, Ernest K. Amankwah, Xiaotao Qu, Ya-Yu Tsai, Heather S. L. Jim, Zhihua Chen, Ann Y. Chen, Jennifer Permuth-Wey, Katja KH. Aben, Hoda Anton-Culver, Natalia Antonenkova, Fiona Bruinsma, Elisa V. Bandera, Yukie T. Bean, Matthias W. Beckmann, Maria Bisogna, Line Bjorge, Natalia Bogdanova, Louise A. Brinton, Angela Brooks-Wilson, Clareann H. Bunker, Ralf Butzow, Ian G. Campbell, Karen Carty, Jenny Chang-Claude, Linda S. Cook, Daniel W. Cramer, Julie M. Cunningham, Cezary Cybulski, Agnieszka Dansonka-Mieszkowska, Andreas du Bois, Evelyn Despierre, Ed Dicks, Jennifer A. Doherty, Thilo Dörk, Matthias Dürst, Douglas F. Easton, Diana M. Eccles, Robert P. Edwards, Arif B. Ekici, Peter A. Fasching, Brooke L. Fridley, Yu-Tang Gao, Aleksandra Gentry-Maharaj, Graham G. Giles, Rosalind Glasspool, Marc T. Goodman, Jacek Gronwald, Patricia Harrington, Philipp Harter, Alexander Hein, Florian Heitz, Michelle A. T. Hildebrandt, Peter Hillemanns, Claus K. Hogdall, Estrid Hogdall, Satoyo Hosono, Anna Jakubowska, Allan Jensen, Bu-Tian Ji, Beth Y. Karlan, Linda E. Kelemen, Mellissa Kellar, Lambertus A. Kiemeney, Camilla Krakstad, Susanne K. Kjaer, Jolanta Kupryjanczyk, Diether Lambrechts, Sandrina Lambrechts, Nhu D. Le, Alice W. Lee, Shashi Lele, Arto Leminen, Jenny Lester, Douglas A. Levine, Dong Liang, Boon Kiong Lim, Jolanta Lissowska, Karen Lu, Jan Lubinski, Lene Lundvall, Leon F. A. G. Massuger, Keitaro Matsuo, Valerie McGuire, John R. McLaughlin, Iain McNeish, Usha Menon, Roger L. Milne, Francesmary Modugno, Kirsten B. Moysich, Roberta B. Ness, Heli Nevanlinna, Ursula Eilber, Kunle Odunsi, Sara H. Olson, Irene Orlow, Sandra Orsulic, Rachel Palmieri Weber, James Paul, Celeste L. Pearce, Tanja Pejovic, Liisa M. Pelttari, Malcolm C. Pike, Elizabeth M. Poole, Harvey A. Risch, Barry Rosen, Mary Anne Rossing, Joseph H. Rothstein, Anja Rudolph, Ingo B. Runnebaum, Iwona K. Rzepecka, Helga B. Salvesen, Eva Schernhammer, Ira Schwaab, Xiao-Ou Shu, Yurii B. Shvetsov, Nadeem Siddiqui, Weiva Sieh, Honglin Song, Melissa C. Southey, Beata Spiewankiewicz, Lara Sucheston, Soo-Hwang Teo, Kathryn L. Terry, Pamela J. Thompson, Lotte Thomsen, Ingvild L. Tangen, Shelley S. Tworoger, Anne M. van Altena, Robert A. Vierkant, Ignace Vergote, Christine S. Walsh, Shan Wang-Gohrke, Nicolas Wentzensen, Alice S. Whittemore, Kristine G. Wicklund, Lynne R. Wilkens, Anna H. Wu, Xifeng Wu, Yin-Ling Woo, Hannah Yang, Wei Zheng, Argyrios Ziogas, Hanis N. Hasmad, Andrew Berchuck, Georgia Chenevix-Trench on behalf of the AOCS management group, Edwin S. Iversen, Joellen M. Schildkraut, Susan J. Ramus, Ellen L. Goode, Alvaro N. A. Monteiro, Simon A. Gayther, Steven A. Narod, Paul D. P. Pharoah, Thomas A. Sellers, Catherine M. Phelan

**Affiliations:** 1 Division of Population Sciences, Department of Cancer Epidemiology, Moffitt Cancer Center, Tampa, Florida, United States of America; 2 Department of Biostatistics and Bioinformatics, Moffitt Cancer Center, Tampa, Florida, United States of America; 3 Department of Oncology, University of Cambridge, Strangeways Research Laboratory, Cambridge, UK; 4 Department of Preventive Medicine, Keck School of Medicine, University of Southern California Norris Comprehensive Cancer Center, Los Angeles, California, United States of America; 5 Clinical and Translational Research Organization, All Children’s Hospital Johns Hopkins Medicine, St Petersburg, Florida, United States of America; 6 Department of Health Outcomes and Behavior, Moffitt Cancer Center, Tampa, Florida, United States of America; 7 Radboud University Medical Center, Radboud Institute for Health Sciences, Nijmegen, The Netherlands; 8 Comprehensive Cancer Center The Netherlands, Nijmegen, The Netherlands; 9 Genetic Epidemiology Research Institute, Center for Cancer Genetics Research and Prevention, University of California Irvine, Irvine, California, United States of America; 10 N.N. Alexandrov National Cancer Centre of Belarus, Minsk, Belarus; 11 Cancer Epidemiology Centre, Cancer Council Victoria, Melbourne, Australia; 12 Cancer Prevention and Control Program, Rutgers Cancer Institute of New Jersey, New Brunswick, New Jersey, United States of America; 13 Department of Obstetrics & Gynecology, Oregon Health & Science University, Portland, Oregon, United States of America; 14 Knight Cancer Institute, Oregon Health & Science University, Portland, Oregon, United States of America; 15 Department of Gynecology and Obstetrics, University Hospital Erlangen, Friedrich-Alexander-University, Erlangen-Nuremberg Comprehensive Cancer Center, Erlangen EMN, Erlangen, Germany; 16 Gynecology Service, Department of Surgery, Memorial Sloan-Kettering Cancer Center, New York, New York, United States of America; 17 Department of Gynecology and Obstetrics, Haukeland University Hospital, Bergen, Norway; 18 Centre for Cancer Biomarkers, Department of Clinical Medicine, University of Bergen, Bergen, Norway; 19 Radiation Oncology Research Unit, Hannover Medical School, Hannover, Germany; 20 Division of Cancer Epidemiology and Genetics, National Cancer Institute, Bethesda, Maryland, United States of America; 21 Canada's Michael Smith Genome Sciences Centre, BC Cancer Agency, Vancouver, Canada; 22 Department of Biomedical Physiology and Kinesiology, Simon Fraser University, Burnaby, Canada; 23 Department of Epidemiology, University of Pittsburgh Graduate School of Public Health, Pittsburgh, Pennsylvania, United States of America; 24 Department of Obstetrics and Gynecology, University of Helsinki and Helsinki University Central Hospital, Helsinki, HUS, Finland; 25 Department of Pathology, Helsinki University Central Hospital, Helsinki, Finland; 26 Cancer Genetics Laboratory Research Division, Peter MacCallum Cancer Centre, St Andrews Place, East Melbourne, Australia; 27 Department of Pathology, the University of Melbourne, Parkville, Australia; 28 Sir Peter MacCallum Department of Oncology, University of Melbourne, Parkville, Victoria, Australia; 29 Department of Gynaecological Oncology, Glasgow Royal Infirmary, Glasgow, United Kingdom; 30 The Beatson West of Scotland Cancer Centre, Glasgow, United Kingdom; 31 German Cancer Research Center (DKFZ), Division of Cancer Epidemiology, Heidelberg, Germany; 32 Division of Epidemiology and Biostatistics, Department of Internal Medicine, University of New Mexico, Albuquerque, New Mexico, United States of America; 33 Obstetrics and Gynecology Center, Brigham and Women's Hospital and Harvard Medical School, Boston, Massachusetts, United States of America; 34 Department of Laboratory Medicine and Pathology, Mayo Clinic, Rochester, Minnesota, United States of America; 35 International Hereditary Cancer Center, Department of Genetics and Pathology, Pomeranian Medical University, Szczecin, Poland; 36 Department of Pathology, The Maria Sklodowska-Curie Memorial Cancer Center and Institute of Oncology, Warsaw, Poland; 37 Department of Gynaecology and Gynaecologic Oncology, Kliniken Essen-Mitte/ Evang. Huyssens-Stiftung/ Knappschaft GmbH, Essen, Germany; 38 Department of Gynaecology and Gynaecologic Oncology, Dr. Horst Schmidt Kliniken Wiesbaden, Wiesbaden, Germany; 39 Division of Gynecologic Oncology, Department of Obstetrics and Gynaecology and Leuven Cancer Institute, University Hospitals Leuven, Leuven, Belgium; 40 Department of Oncology, The Centre for Cancer Epidemiology, University of Cambridge, Strangeways Research Laboratory, Cambridge, United Kingdom; 41 Department of Epidemiology, The Geisel School of Medicine Hanover, New Hampshire, United States of America; 42 Program in Epidemiology, Division of Public Health Sciences, Fred Hutchinson Cancer Research Center, University of Washington, Seattle, Washington, United States of America; 43 Gynaecology Research Unit, Hannover Medical School, Hannover, Germany; 44 Department of Gynecology, Jena University Hospital, Friedrich Schiller University, Jena, Germany; 45 Centre for Cancer Genetic Epidemiology, Department of Oncology, University of Cambridge, Cambridge, United Kingdom; 46 Faculty of Medicine, University of Southampton, Southampton, United Kingdom; 47 Ovarian Cancer Center of Excellence, Womens Cancer Research Program, Magee-Womens Research Institute and University of Pittsburgh Cancer Institute, Pittsburgh, Pennsylvania, United States of America; 48 Institute of Human Genetics, Friedrich-Alexander-University Erlangen-Nuremberg, Comprehensive Cancer Center Erlangen-Nuremberg, Erlangen, Germany; 49 University of California at Los Angeles, David Geffen School of Medicine, Department of Medicine, Division of Hematology and Oncology, Los Angeles, California, United States of America; 50 Biostatistics and Informatics Shared Resource, University of Kansas Medical Center, Kansas City, Kansas, United States of America; 51 Department of Epidemiology, Shanghai Cancer Institute, Shanghai, China; 52 Women's Cancer, Institute for Women's Health, University College London, London, United Kingdom; 53 Centre for Epidemiology and Biostatistics, School of Population and Global Health, The University of Melbourne, Melbourne, Australia; 54 Cancer Prevention and Control, Samuel Oschin Comprehensive Cancer Institute, Cedars-Sinai Medical Center, Los Angeles, California, United States of America; 55 Community and Population Health Research Institute, Department of Biomedical Sciences, Cedars-Sinai Medical Center, Los Angeles, California, United States of America; 56 Department of Epidemiology, The University of Texas MD Anderson Cancer Center, Houston, Texas, United States of America; 57 Molecular Unit, Department of Pathology, Herlev Hospital, University of Copenhagen, Copenhagen, Denmark; 58 Department of Virus, Lifestyle and Genes, Danish Cancer Society Research Center, Copenhagen, Denmark; 59 Department of Preventive Medicine, Kyushu University Faculty of Medical Science, Nagoya, Aichi, Japan; 60 Women's Cancer Program at the Samuel Oschin Comprehensive Cancer Institute, Cedars-Sinai Medical Center, Los Angeles, California, United States of America; 61 Department of Public Health Sciences, Medical University of South Carolina, Charleston, South Carolina, United States of America; 62 Department of Gynaecology, Rigshospitalet, University of Copenhagen, Copenhagen, Denmark; 63 The Juliane Marie Centre, Department of Gynecology, Righospitalet, University of Copenhagen, Copenhagen, Denmark; 64 Vesalius Research Center, VIB, Leuven, Belgium; 65 Laboratory for Translational Genetics, Department of Oncology, University of Leuven, Belgium; 66 Cancer Control Research, BC Cancer Agency, Vancouver, Canada; 67 Department of Cancer Prevention and Control, Roswell Park Cancer Institute, Buffalo, New York, United States of America; 68 College of Pharmacy and Health Sciences, Texas Southern University, Houston, Texas, United States of America; 69 Department of Obstetrics and Gynaecology, University Malaya Medical Centre, University Malaya, Kuala Lumpur, Malaysia; 70 Department of Cancer Epidemiology and Prevention, M. Sklodowska-Curie Memorial Cancer Center and Institute of Oncology, Warsaw, Poland; 71 Department of Gynecologic Oncology, The University of Texas MD Anderson Cancer Center, Houston, Texas, United States of America; 72 Department of Gynaecology, Radboud University Medical Center, Nijmegen, The Netherlands; 73 Department of Health Research and Policy—Epidemiology, Stanford University School of Medicine, Stanford, California, United States of America; 74 Public Health Ontario, Toronto, Canada; 75 Institute of Cancer Sciences, University of Glasgow, Wolfson Wohl Cancer research Centre, Beatson Institute for Cancer Research, Glasgow, UK; 76 Women's Cancer Research Program, Magee-Women's Research Institute and University of Pittsburgh Cancer Institute, Pittsburgh, Pennsylvania, United States of America; 77 Department of Obstetrics, Gynecology and Reproductive Sciences, University of Pittsburgh School of Medicine, Pittsburgh, Pennsylvania, United States of America; 78 Department of Pathology, Rigshospitalet, University of Copenhagen, Copenhagen, Denmark; 79 The University of Texas School of Public Health, Houston, Texas, United States of America; 80 Department of Gynecologic Oncology, Roswell Park Cancer Institute, Buffalo, New York, United States of America; 81 Department of Epidemiology and Biostatistics, Memorial Sloan-Kettering Cancer Center, New York, New York, United States of America; 82 Department of Community and Family Medicine, Duke University Medical Center, Durham, North Carolina, United States of America; 83 Department of Epidemiology, University of Michigan School of Public Health,Ann Arbor, Michigan, United States of America; 84 Channing Division of Network Medicine, Brigham and Women's Hospital and Harvard Medical School, Boston, Massachusetts, United States of America; 85 Department of Epidemiology, Harvard School of Public Health, Boston, Massachusetts, United States of America; 86 Department of Chronic Disease Epidemiology, Yale School of Public Health, New Haven, Connecticut, United States of America; 87 Department of Gynecology-Oncology, Princess Margaret Hospital, and Department of Obstetrics and Gynecology, Faculty of Medicine, University of Toronto, Toronto, Ontario, Canada; 88 Department of Health Research and Policy- Epidemiology, Stanford University School of Medicine, Stanford, California, United States of America; 89 Institut für Humangenetik, Wiesbaden, Germany; 90 Epidemiology Center and Vanderbilt, Ingram Cancer Center, Vanderbilt University School of Medicine, Nashville, Tennessee, United States of America; 91 Cancer Epidemiology Program, University of Hawaii Cancer Center, Hawaii, United States of America; 92 Department of Gynecologic Oncology, The Maria Sklodowska-Curie Memorial Cancer Center and Institute of Oncology, Warsaw, Poland; 93 Cancer Research Initiatives Foundation, Sime Darby Medical Center, Subang Jaya, Malaysia; 94 University Malaya Cancer Research Institute, University Malaya Medical Centre, University Malaya, Kuala Lumpur, Maylaysia; 95 Department of Health Science Research, Division of Biomedical Statistics and Informatics, Mayo Clinic, Rochester, Minnesota, United States of America; 96 Vanderbilt Epidemiology Center, Vanderbilt University School of Medicine, Nashville, Tennessee, United States of America; 97 Department of Obstetrics and Gynecology, Duke University Medical Center, Durham, North Carolina, United States of America; 98 QIMR Berghofer Medical Research Institute, Brisbane, Australia; 99 Peter MacCallum Cancer Centre, East Melbourne, Australia; 100 Department of Statistics, Duke University, Durham, North Carolina, United States of America; 101 Cancer Prevention, Detection & Control Research Program, Duke Cancer Institute, Durham, North Carolina, United States of America; 102 Department of Health Science Research, Division of Epidemiology, Mayo Clinic, Rochester, Minnesota, United States of America; 103 Women's College Research Institute, University of Toronto, Toronto, Ontario, Canada; 104 Department of Public Health and Primary Care, University of Cambridge, Cambridge, United Kingdom; Florida International University, UNITED STATES

## Abstract

**Background:**

Defective cellular transport processes can lead to aberrant accumulation of trace elements, iron, small molecules and hormones in the cell, which in turn may promote the formation of reactive oxygen species, promoting DNA damage and aberrant expression of key regulatory cancer genes. As DNA damage and uncontrolled proliferation are hallmarks of cancer, including epithelial ovarian cancer (EOC), we hypothesized that inherited variation in the cellular transport genes contributes to EOC risk.

**Methods:**

In total, DNA samples were obtained from 14,525 case subjects with invasive EOC and from 23,447 controls from 43 sites in the Ovarian Cancer Association Consortium (OCAC). Two hundred seventy nine SNPs, representing 131 genes, were genotyped using an Illumina Infinium iSelect BeadChip as part of the Collaborative Oncological Gene-environment Study (COGS). SNP analyses were conducted using unconditional logistic regression under a log-additive model, and the FDR q<0.2 was applied to adjust for multiple comparisons.

**Results:**

The most significant evidence of an association for all invasive cancers combined and for the serous subtype was observed for SNP rs17216603 in the iron transporter gene *HEPH* (invasive: OR = 0.85, P = 0.00026; serous: OR = 0.81, P = 0.00020); this SNP was also associated with the borderline/low malignant potential (LMP) tumors (P = 0.021). Other genes significantly associated with EOC histological subtypes (p<0.05) included the *UGT1A* (endometrioid), *SLC25A45* (mucinous), *SLC39A11* (low malignant potential), and *SERPINA7* (clear cell carcinoma). In addition, 1785 SNPs in six genes (*HEPH*, *MGST1*, *SERPINA*, *SLC25A45*, *SLC39A11* and *UGT1A*) were imputed from the 1000 Genomes Project and examined for association with INV EOC in white-European subjects. The most significant imputed SNP was rs117729793 in *SLC39A11* (per allele, OR = 2.55, 95% CI = 1.5-4.35, p = 5.66x10^-4^).

**Conclusion:**

These results, generated on a large cohort of women, revealed associations between inherited cellular transport gene variants and risk of EOC histologic subtypes.

## Introduction

Epithelial ovarian carcinoma (EOC) is the second-most common gynecological malignancy and the leading cause of gynecological cancer-related mortality in the United States and other developed nations [1]. Early stage EOC is accompanied by vague, non-specific symptoms and is difficult to detect. As yet, no EOC screening or early detection strategies have been proven useful in general population [2]. As a consequence, approximately 60% of cases are diagnosed at advanced stages (III-IV), with 5-year overall survival of less than 20% [3]. Given these grim statistics, improved understanding of the etiology of EOC is critical to reducing the associated morbidity and mortality.

An inadequate understanding of genetic and biological etiology of EOC has limited the ability to detect and treat this disease effectively. Disruptions in cellular transport, lead to abnormal levels of trace elements (iron, zinc and copper), hormones and small molecules which impact the expression of key regulatory genes. Aberrant expression of transmembrane transport genes has been associated with increased risk, as well as aggressiveness, of a number of cancers including breast [4–7], prostate [8], liver [9], colorectal and colon [10–12], thyroid [13] and neuroblastoma [14]. Iron is essential for erythropoiesis [15] and the function of the mitochondrial respiratory chain where it plays a key role in electron transport, in the form of iron-sulphur clusters or in heme centers [16]. However, excess iron and copper have been reported to promote the formation of reactive oxygen species (ROS) which damage cellular DNA and support cancer growth [17]. Hydrogen peroxide generated as a byproduct in the mitochondrial respiratory chain, can react with iron or copper and form hydroxyl radicals that are extremely reactive and damaging to the genome [18]. Consistent with these data, transport of the three most abundant transition metals in humans–iron, zinc and copper–has been linked to the etiology of colorectal and liver cancer [9,19] and prostate cancer [20].

Despite the growing evidence that cellular transport processes influence cancer risk, the association of germline genetic variation in cellular transport genes and EOC risk has not been well studied. As histologic subtypes of EOC differ in clinical behavior and biologic and genetic origin [21], we hypothesized that single nucleotide polymorphisms (SNPs) in cellular transport genes are associated with EOC risk and vary by histopathology. This study examined the association of 279 SNPs in 131 cellular transport genes and EOC risk in an international collaboration that included 18,174 case and 26,134 control subjects.

## Materials and Methods

### Sample and Procedure

The discovery set included DNA samples from 3,761 EOC case subjects and 2,722 control subjects in two ovarian cancer Genome Wide Association Studies (GWAS) in North America and the United Kingdom (UK). Details of these studies have been previously published [22]. In brief, the North American study was comprised of four case-control studies genotyped using the Illumina 610-quad Beadchip Array (1,814 case subjects and 1,867 control subjects) as well as a single case-control study genotyped on the Illumina 317K and 370K arrays (133 case subjects and 142 control subjects). The UK study was comprised of four case-only studies genotyped on the Illumina 610-quad Beadchip Array and two common control sets genotyped on the Illumina 550K array (1,814 case subjects and 713 control subjects).

The replication sample consisted of DNA samples from 14,525 women with invasive EOC and 23,447 control women with European ancestry from 43 sites in the Ovarian Cancer Association Consortium (OCAC). Samples from an additional 1,747 participants with tumors of low malignant potential were also analyzed. Details of the sample QC are provided in Pharoah et al., [22]. This replication set include 89 SER cases and 200 controls of African ancestry, and 249 SER cases and 1574 controls of Asian ancestry.

All research involving human participants has been approved by each study site’s local Institutional Review Board (IRB) according to the principles expressed in the Declaration of Helsinki. Written informed consent was obtained from all of the participants. The permission to use of the data in this study was granted by the University of South Florida (IRB#: Pro00000249).

### Gene and SNP Selection

Gene (NCBI) [23], BioCarta [24], GenomeNet [25] and other relevant gene/pathway databases were used to select the candidate genes for inclusion in the transport pathway. Specifically, the search yielded 131 genes involved in transport of trace elements, ions, hormones and small molecules. A total of 5202 SNPs in the selected genes were identified from the Human-610 Quad BeadChip (Illumina). Those SNPs were genotyped in the four ovarian cancer GWAS studies (US GWAS, UK GWAS, COGS and Mayo clinic). The final selection of transport gene SNPs for genotyping in the replication stage was informed according to lowest p-values (cut off p<0.01 was used) by ranking of minimal p-values across four sets of results in the discovery set: 1) North American all histologies, 2) North American serous histology, 3) combined GWAS meta-analysis all histologies, and 4) combined GWAS meta-analysis serous histology. Additional functional SNPs in these genes were also included. In total 299 SNPs were included on the COGS chip of which 279 SNPs (in 131 genes) passed QC (described in detail in Pharoah et al., [22]).

### Imputation Analyses

These analyses were based on imputed genotypes from the four ovarian cancer GWAS studies (US GWAS, UK GWAS, COGS and Mayo clinic) with a total of 15,398 invasive EOC case subjects and 30,816 control subjects of white-European ancestry. Imputation of each dataset into the 1000 Genomes Project was performed using IMPUTE2 software [26]. We used the 1000 Genomes Project v3 as the reference with pre-phasing of the data using SHAPEIT [27].

### Statistical Analysis

For the discovery set, the North American and UK studies were analyzed separately and four sets of results: 1) North American all histologies, 2) North American serous histology, 3) combined GWAS meta-analysis all histologies, and 4) combined GWAS meta-analysis serous histology were conducted. SNP analyses were performed using unconditional logistic regression under a log-additive model. The last two sets were analyzed using the fixed effects meta-analysis. These analyses were carried out in PLINK [28] by combining results across studies by using the Mantel–Haenszel method [29].

For the replication set, demographic and clinical characteristics of case subjects and control subjects were compared using t-test for continuous variables and chi-square test for categorical variables. Unconditional logistic regression was used to evaluate associations between SNPs and ovarian cancer risk. SNPs were modeled using number of minor alleles as ordinal variables (log-additive model). Per-allele log odds ratios and their 95% confidence intervals were estimated. All analyses were done separately by race groups.

In order to adjust for population substructure, intercontinental ancestry was assigned based on genotype frequencies for European, Asian, and African populations using LAMP software [30] Subjects with greater than 90 percent European ancestry were defined as European; those with greater than 80 percent Asian and African ancestry were defined as being Asian and African, respectively. The set of 37,000 unlinked markers was applied to perform principal-components analysis within each major population subgroup [31].The number of principal components was based on the inflection position of the principal components scree plot. The models for white-European subjects were adjusted for study site and for the first five principal components. For the African American (AA) and Asian (AS) study subjects, the first one and five principal components were included in the models, respectively.

We evaluated associations between candidate SNPs and risk of all invasive EOC (INV), each of the four main histological subtypes (serous (SER); endometroid (END); clear cell (CC); and mucinous (MUC)), and tumors of low malignant potential (LMP). Odds ratios for each histologic subtype were estimated by comparing cases of each subtype to all controls as reference. False discovery rate (FDR) q-value was applied for adjusting multiple comparisons [32]. Associations with a p value < .05 and a false discovery rate (FDR) q-value < .20 were considered statistically significant.

For the imputation set analyses, the meta-analysis using an in-house program written in C++ was carried out for combining results across studies. A fixed effect meta-analysis was used, with the log odds ratio being the estimate for each study and the standard error of this estimate determining the weighting. For each SNP, only the studies with valid estimates for that SNP (i.e. r2 > 0.25) were used in the meta-analysis calculation.

## Results

Sample characteristics are described in the S1 Table. As expected, significant differences were observed between case and control subjects on EOC risk factors including age, family history of ovarian cancer, age at menarche, body mass index (BMI), history of oral contraceptive use, and number of full term births (p-values<0.05). The proportion of tumors of SER (57.6%) was higher than that of other subtypes (14.2% END, 7.1% CC, 6.5% MUC, and 14.6% other), which is typical for white-European populations.

Among the 279 cellular transport SNPs genotyped in the replication set, 81 SNPs in 48 genes showed nominally significant (p<0.05) associations with at least one histological subtype (S2 Table). All invasive cancers combined (INV), LMP and the four main histological subtypes (SER [n = 8,369]; END [n = 2,067]; CC [n = 1,024]; and MUC [n = 943]) were analyzed.

The strongest evidence of an association for INV EOC was observed for SNP rs17216603 in the iron transporter gene *HEPH* (OR = 0.85, 95%CI = 0.77–0.93, P = 2.55x10^-4^; FDR q-value = 0.065), which was also the most significant SNP associated with SER (P = 1.99x10^-4^; 95%CI = 0.73–0.91, FDR q-value = 0.054), and LMP subtypes (P = 0.0206) (Table 1). The most significant association for END EOC was rs11563251 within the *UGT1A* gene cluster (OR = 0.82, 95%CI = 0.73–0.92, P = 6.59x10^-4^; FDR q-value = 0.177). Only these two SNPs were associated with q-values <0.20. The most significant association for MUC subtype was rs681309, near the *SLC25A45* gene (OR = 0.89, 95%CI = 0.81–0.97, P = 0.012). This SNP was also associated with the END subtype (P = 0.0035) and INV EOC (P = 0.029). Association with rs9908917, in the intron of *SLC39A11*, was observed for LMP cases (OR = 0.85, 95%CI = 0.77–0.93, P = 3.9x10^-4^). This SNP was also associated with the SER subtype (P = 0.0123) and INV EOC (P = 0.0144). The SNP rs1804495 in *SERPINA7* was associated with SER, MUC, CC, INV and LMP (P<0.05), but not END (P>0.05).

**Table 1:** The most significant SNPs in the transport pathway genes and risk of EOC by histology, invasiveness, and race/ethnicity^1^.

GENE SNP	INV	LMP	SER	CC	END	MUC	Asian (SER)	African-American (SER)
*HEPH*rs17216603	**0.85 (0.77–0.93); 2.55x10** ^**-4**^ **; q = 0.063**	**0.78 (0.63–0.97); 0.021;**	**0.81 (0.73–0.91); 1.99x10** ^**-4**^ **; q = 0.054**	0.77 (0.58–1.02); 0.07	0.9 (0.74–1.08); 0.26	0.92 (0.7–1.21); 0.56	**1.45 (1.15–1.83) 0.0019**	0.76 (0.15–3.86); 0.74
*SLC39A11*rs9908917	**0.95 (0.92–0.99); 0.014**	**0.85 (0.77–0.93); 3.9x10** ^**-4**^	**0.94 (0.9–0.99); 0.010**	1.01 (0.91–1.13); 0.82	0.94 (0.87–1.02); 0.16	0.98 (0.87–1.00); 0.7	**1.25 (1.01–1.56); 0.049**	1.23 (0.85–1.77); 0.27
*SERPINA7*Rs1804495	**1.05 (1.00–1.1); 0.042**	**1.14 (1.03–1.27); 0.016**	**1.06 (1.00–1.12); 0.045**	**1.21 (1.06–1.39); 0.0042**	1.06 (0.96–1.17); 0.28	**0.85 (0.73–1.00); 0.045**	*0*.*81 (0*.*65–1*.*00); 0*.*05*	1.11 (0.75–1.65); 0.6
*SLC25A45*Rs681309	**0.97 (0.94–1.00); 0.029**	1.04 (0.97–1.12); 0.27	1 (0.96–1.04); 0.99	0.98 (0.89–1.07); 0.59	**0.91 (0.85–0.97); 0.0035**	**0.89 (0.81–0.97); 0.012**	**0.78 (0.63–0.98); 0.033**	0.87 (0.6–1.26); 0.41
*UGT1A* Rs11563251	*0*.*95 (0*.*91–1*.*00); 0*.*05*	1.08 (0.96–1.2); 0.21	0.95 (0.89–1.01); 0.07	1.03 (0.89–1.18); 0.73	**0.82 (0.74–0.92); 6.8x10** ^**-4**^ **; q = 0.18**	1.06 (0.91–1.22); 0.49	0.73 (0.5–1.06); 0.1	1.22 (0.85–1.25); 0.28
*MGST1* Rs6488840	**0.96 (0.93–1.00); 0.048**	1.0 (0.92–1.09); 0.9	**0.95 (0.91–1.00); 0.042**	0.96 (0.86–1.07); 0.41	1 (0.92–1.08); 0.95	0.96 (0.86–1.08); 0.48	0.54 (0.25–1.18); 0.12	**0.55 (0.37–0.82); 0.0035**

^1^ INV: all invasive EOC combined; LMP: low malignant potential / borderline tumors; SER: serous; CC: clear cell; End: endometrioid; Muc: mucinous. Statistically significant associations are indicated in bold (P<0.05). Data format is the following: OR (95% CI); p-value; FDR q-value (white-European women). Only significant FDRs (q<0.2) are shown (*HEPH*: INV and SER*; UGT1A*: End).

### Imputed Variants

In total, 1785 imputed SNPs in six genes (*HEPH*, *MGST1*, *SERPINA*, *SLC25A45*, *SLC39A11* and *UGT1A*) were examined for association with INV EOC in white-European subjects only. From these, 274 SNPs were found with p-value<0.05 (S3 Table). Across all six genes, the most significant imputed SNP was rs117729793 in *SLC39A11* (per allele, OR = 2.55, 95% CI = 1.5–4.35, p = 5.66x10^-4^). Interestingly, 190 of 274 (~70%) imputed SNPs with p-values < 0.05 were located in or near *SLC39A11*.

### Results in women of African-American (AA) and Asian (AS) ethnicities

We conducted exploratory analyses for other ethnicities and SER EOC. Fourteen SNPs showed significant associations in AS and AA women. Six of the SNPs in the AS women were also significant in the white-European women, compared to two of the 14 SNPs in the AA women. The top SNP in women of Asian ancestry (rs17216603 in *HEPH*) was shared with the white-European women. The *SLC25A45* rs681309 was also shared. *SERPINA7* rs1804495 was borderline significant (P = 0.0503), perhaps due to a small sample size. The most significant association in women of AA ancestry was noted at the SNP rs6488840 near to the microsomal glutathione S-transferase 1 (*MGST1*) gene. In our study, *MGST1* rs6488840 was associated with statistically significantly reduced SER EOC risk in women of AA ancestry (OR = 0.55; P = 0.0035). This SNP was of borderline significance in women of white-European (OR = 0.95; P = 0.042), but not Asian (P>0.05), ancestry. In the groups of AS and AA women, no SNPs had FDR q-value <0.20. Results for the top hits across women of different ancestries are presented in Table 2.

**Table 2:** Top SNPs associated with SER EOC across racial groups.

Race	White-European			Asian			African American		
GENE SNP	MAF^1^	p-value^2^	OR (95% CI)^3^	MAF^1^	p-value^2^	OR (95% CI) ^3^	MAF^1^	p-value^2^	OR (95% CI) ^3^
***HEPH* rs17216603**	A = 0.03	**2x10** ^**-4**^	**0.81 (0.73–0.91)**	A<0.01	**0.002**	**1.45 (1.15–1.83)**	A<0.01	0.74	0.76 (0.15–3.86)
***SLC39A11* rs9908917**	T = 0.13	**0.010**	**0.94 (0.9–0.99)**	T = 0.2	**0.049**	**1.25 (1.0–1.56)**	T<0.01	0.27	1.2 (0.85–1.78)
***SERPINA7* rs1804495**	A = 0.12	**0.045**	**1.06 (1–1.12)**	A = 0.23	0.050	0.8 (0.65–1.0)	A = 0.1	0.60	1.1 (0.75–1.65)
***SLC25A45* rs681309**	G = 0.49	0.990	1 (0.96–1.04);	G = 0.26	**0.033**	**0.78 (0.63–0.98)**	G = 0.1	0.41	0.8 (0.57–1.26)
***UGT1A* rs11563251**	T = 0.12	0.074	0.95 (0.89–1.01)	T = 0.07	0.100	0.7 (0.5–1.06)	T = 0.42	0.281	1.2 (0.85–1.75)
***MGST1* rs6488840**	C = 0.2	**0.042**	**0.95 (0.91–1)**	A = 0.42	0.121	0.54 (0.25–1.18)	C = 0.35	**0.004**	**0.55 (0.37–0.82)**

^1^ MAF, minor allele and its frequency

^2^ p-value <0.05 are in bold

^3^ Odds ratio, 95% confidence interval

## Discussion

**Fig 1:**
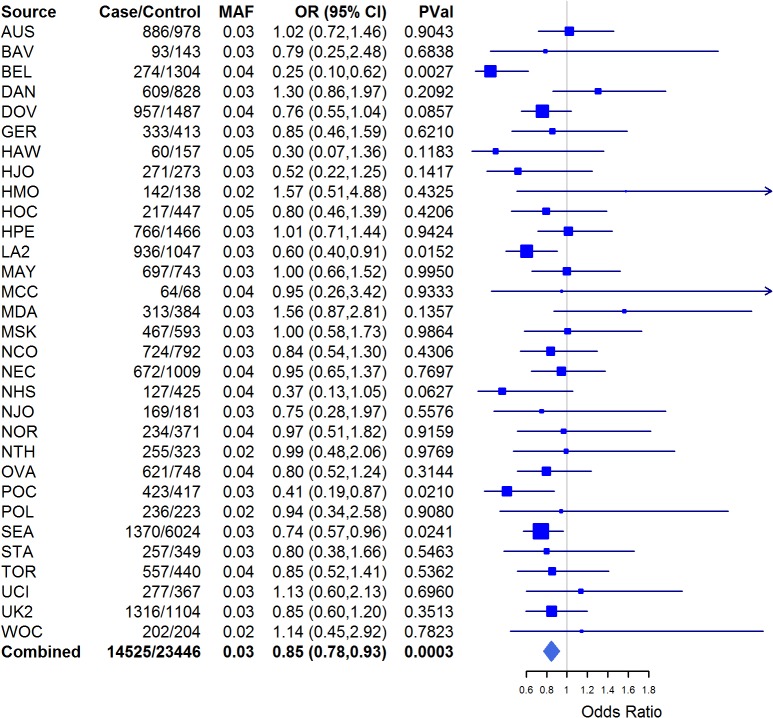
Forest plot for *HEPH* rs17216603 across studies. Squares represent the estimated per-allele odds ratio (OR) for each study. Lines indicate the 95% confidence intervals. Diamond represents the OR estimate and confidence limits. Invasive EOC risk in women of white-European descent only; MAF in controls.

The development and progression of ovarian cancer is accompanied by aberrant cellular metabolism [33]. Central to cellular metabolic processes are the transport of trace elements and hormones through cellular and nuclear membranes. In this study, we aimed to elucidate whether germline SNPs in cellular transport genes were associated with EOC risk and histopathologic subtype. We detected nominal associations (P<0.05) with 81 SNPs and EOC risk in at least one of the histopathologic subtypes (S3 Table). Associations were noted with rs17216603 in *HEPH* and SER, INV and LMP subgroups as well as in SER and INV cases in women of white-European and Asian ancestries. The Hephaestin (*HEPH*) gene encodes a transmembrane copper-dependent ferroxidase (HEPH protein) responsible for dietary iron transport from intestinal enterocytes into the blood stream [34–36]. *HEPH* catalyzes ferrous (F^2+^) iron reoxidation to its ferric (F^3+^) state [37–38] that can be utilized by the body. The role of iron homeostasis in cancer progression is yet to be fully understood; however depletion of iron stores in cells induces cell cycle arrest and apoptosis, limits the rate of DNA synthesis, and down-regulates expression of various potentially carcinogenic kinases such as cyclins and cyclin-dependent kinases [39]. Additionally, iron is known to facilitate generation of mutagenic reactive oxygen species (ROS) that may drive cancer development and progression [40] as has been observed in colorectal cancer [41]. *In silico* analysis of *HEPH* rs17216603 combining results from Snpnexus, SNPinfo and Annovar [42–44] showed that this variant results in the substitution of Alanine at residue 598 with Threonine and may lead to reduction or loss of *HEPH* function. Functional analyses of this SNP and gene will be needed to clarify the impact of this finding. There was no evidence of MAF heterogeneity because the MAF range of rs17216603 across the studies is 2–5%. This SNP was significantly associated with Invasive EOC risk in women of white-European descent (P = 0.0003) for the combined results (Fig 1).

The SNP rs9908917 lies within an intron of the *SLC39A11* gene, and was associated with SER, all INV and LMP EOC. In addition, of the 1785 SNPs in six genes (*HEPH*, *MGST1*, *SERPINA*, *SLC25A45*, *SLC39A11* and *UGT1A*) imputed from the 1000 Genomes Project and examined for association with INV EOC in white-European subjects, the most significant imputed SNP was rs117729793 in *SLC39A11* (per allele, OR = 2.55, 95% CI = 1.5–4.35, p = 5.66x10^-4^). In The Cancer Genome Atlas (TCGA) data [45], expression of *SLC39A11* was significantly higher in ovarian tumors compared to normal tissues (P = 9.99x10^-8^). Taken together, these data highlight a potential role for this gene in EOC pathogenesis. The solute carrier family 39, member 11 (*SLC39A11*) is a poorly studied gene belonging to a family of metal ion transporters. *SLC39A11* may act as a zinc-influx transporter, although the exact functions of the A11 gene have yet to be experimentally established [46]. Other members of the *SLC39* family transport metal ions, such as iron, copper, cadmium and manganese [47].

SNP rs681309, in the intergenic region near *SLC25A45*, showed significant associations with all INV, MUC, and END and was the most significant SNP among the MUC subtype. The solute carrier family 25 (mitochondrial carrier; adenine nucleotide translocator), member 45 (*SLC25A45*) belongs to the family of membrane proteins that catalyze the transport of solutes across the inner mitochondrial membrane [48]. While substrates for the *SLC25* family carriers include ADP/ATP, amino acids, malate, ornithine, and citruline [49], the predominant substrate(s) for *SLC23A45* have not yet been characterized [50], although sequence similarity to *SLC25A29* suggests that this protein may be involved in the transport of long-chain fatty acids such as palmitoylcarnitine and acylcarnitine [51]. *SLC25A45* is expressed in skeletal muscle, intestine, brain, and testis and is downregulated during ovarian cancer progression [50]. Taken together, these data suggest that additional studies are warranted on the role of *SLC25A4* in particular, as well as mitochondria in general, in EOC etiology.

The SNP, rs1804495 in *SERPINA7* was associated with all INV, SER, MUC, CC and LMP subtypes, and was the most statistically significant association among the CC subtype. The serpin peptidase inhibitor, clade A (alpha-1 antiproteinase, antitrypsin), member 7 (*SERPINA7*), also known as thyroxine-binding globulin (TBG), is a protein that binds thyroid hormones thyroxin (T4) and 3,5,3’-triiodothyronine (T3) in circulation [52]. Numerous mutations in *SERPINA7* have been identified [53–54] leading to partially or completely absent TBG function. The hallmark of TBG deficiency is abnormally low T3 and T4 combined with normal thyroid stimulating hormone (TSH) values [55]. The specific role of *SERPINA7* in cancer etiology has not been established; however, thyroid hormones may support cancer growth [56]. Thus, it is conceivable that altered TBG production over many years may modulate growth of early-stage ovarian cancer cells. The index SNP, rs1804495, is coding, but the resulting missense change is predicted to have neutral impact on the protein function, suggesting a linked variant may be the causal allele at this locus. We note that in our analyses, rs1804495 confers statistically significantly increased risk for LMP, INV, SER and CC, but decreased risk for mucinous EOC. This observation highlights observations from previously published studies that reveal differences between MUC and other EOCs [57–59].

The most significant association among the END subtype was with *UGT1A1* rs11563251. UDP glucuronosyltransferase 1 family, polypeptide A cluster (*UGT1A*) represents a complex locus which encodes nine human UDP-glucuronosyltransferases. UDP-glucuronosyltransferase (*UGT*) enzymes are localized to endoplasmic reticulum (ER) and catalyze glucuronidation, which is involved in the elimination of bilirubin, steroids, bile acids, toxic dietary components, and several drugs, including morphine, and irinotecan [60–61]. Genetic variation in *UGT1A1* is involved in inherited disorders of bilirubin metabolism such as Crigler-Najjar syndrome, which is manifested in complete absence (type 1) or diminished (types 2–3) bilirubin glucuronidation and resulting impaired bilirubin excretion. Previously, the UGT1A7*3 allele exhibited modestly significant association with colorectal cancer (OR = 2.39; P = 0.02) [57], lung cancer [62], endometrial cancer [63] and pancreatic cancer (OR = 1.98; *P* = 0.003) [64] with a particularly strong association in smokers with pancreatic carcinoma who were younger than 55 years (OR = 4.7; *P* = 0.0009), suggesting the magnitude of the observed associations may be modified by environmental interactions. Down-regulation of *UGT1A* appears to be an early event in carcinogenesis [65]; it is postulated that constitutive expression of *UGT1A* family genes in normal mucosa protects organs from carcinogens released in the bladder or absorbed from the diet in the colon. The rs11563251 variant lies within the 3’UTR of the *UGT1A1*,-*A6* and–*A10* genes, and so could feasibly impact the RNA stability of these transcripts. Alternatively, since this SNP also lies within intronic sequences of other *UGT1A* genes, this SNP could possibly be involved in *cis*-regulation of expression of one or more genes in this cluster.

In this study we conducted exploratory analyses in AS and AA subjects. However, the power to detect associations in women of non-European ancestries was limited due to small sample size and only the SER subtype of EOC was investigated for risk associations. The top SNPs in the AS ancestry group (rs17216603 in *HEPH* and rs1552846 in *SLC39A11*) were also significant in white-European women. In women of AA ancestry, the most significant SNP rs6488840 (P = 0.0035) was close to the microsomal glutathione S-transferase 1 (*MGST1*) gene, which encodes a protein that catalyzes the conjugation of glutathione to electrophiles and the reduction of lipid hydroperoxides. This protein is localized to the endoplasmic reticulum and outer mitochondrial membrane where it is thought to protect these membranes from oxidative stress. The product of this gene is involved in cellular defenses against toxic, carcinogenic, and pharmacologically active electrophilic compounds [66]. *MGST1* overexpression has been demonstrated in various cancers (e.g., prostate cancer and lung cancer [67–68]) and has been associated with high metastatic potential and chemoresistance [69]. *MGST1* is abundantly expressed in EOC primary tumors, metastases and effusions [66]. Other significant SNPs in women of AA ancestry include various members of the SLC39 family: *SLC39A11* (rs9905659 and rs16977431) and *SLC39A8* (rs233807).

The main strength of our study is a large sample size of white-European women that afforded sufficient statistical power to detect modest risk differences. Weaknesses, however, include the lack of functional or metabolic studies to establish biological significance of the observed associations. Another weakness is a small sample size in AA and AS women. The contribution of genetic and/or biological differences to EOC among different ethnic groups is unclear. However, because ovarian cancer health disparities are observed along the whole continuum of the disease globally and in the U.S. [70], this topic is without a doubt important and deserves its own dedicated studies.

In summary, we have found that genetic variation in transmembrane transport genes appear to be associated with EOC risk across various histologic subtypes (Table 1). These data suggest that disruptions in cellular transport (trace elements, hormones and small molecules) may play roles in EOC pathogenesis. Functional and metabolic studies are needed to support these findings.

## Supporting Information

S1 Table Demographic and clinical characteristics of white-European case subjects with invasive disease (n = 14,525) and controls (n = 23,447) in the COGS study. (DOCX)

S2 Table Transport pathway SNPs statistically significantly (p<0.05) associated with at least one histopathologic EOC subtype in white-European women. Significant associations are bolded.
(DOCX)

S3 Table Imputed SNPs significantly (p<0.05) associated with INV EOC in white-European women. SNPs are sorted by p-values.
(DOCX)
