# Supplementary material for: Common Genetic Variation In Cellular Transport Genes and Epithelial Ovarian Cancer (EOC) Risk
Source: PLoS One. 2015 Jun 19;10(6):e0128106. doi: 10.1371/journal.pone.0128106 (PMC4474865; doi:10.1371/journal.pone.0128106)
Supplement: S1 Table — (DOCX) [file pone.0128106.s001.docx]

**Supplementary Table 1**.

| Characteristics | Controls (n=23,447)  N (%) | Invasive Cases (n=14,525)  N (%) | p-value^2^ |
| --- | --- | --- | --- |
| Age (years)  Mean ± SD | 55.6± 11.9 | 58.1± 11.3 | <.0001 |
| <40 | 2027 (8.7) | 748 (5.2) | <.0001 |
| 40-49 | 4771 (20.6) | 2544 (17.6) |  |
| 50-59 | 7403 (31.9) | 4537 (31.3) |  |
| 60-69 | 6098 (26.3) | 4324 (29.8) |  |
| ≥70 | 2892 (12.5) | 2343 (16.2) |  |
| Family history of ovarian cancer^1^ |  |  |  |
| No | 15425 (92.0) | 8634 (82.4) | <.0001 |
| Yes | 1351 ( 8.0) | 1849 (17.6) |  |
| Age at menarche (years) |  |  |  |
| Mean ± SD | 12.9± 1.7 | 12.8± 1.6 | 0.0314 |
| <12 | 3128 (19.3) | 1856 (19.2) | 0.0772 |
| 12 | 3602 (22.2) | 2257 (23.4) |  |
| 13 | 4357 (26.9) | 2621 (27.1) |  |
| ≥14 | 5112 (31.6) | 2923 (30.3) |  |
| Body mass index (kg/m^2^) |  |  |  |
| <25 | 3834 (48.2) | 2528 (45.1) | 0.0006 |
| 25-29 | 2332 (29.3) | 1681 (30.0) |  |
| ≥30 | 1797 (22.6) | 1396 (24.9) |  |
| Oral contraceptive use |  |  |  |
| No | 6136 (37.5) | 4203 (43.7) | <.0001 |
| Yes | 10230 (62.5) | 5419 (56.3) |  |
| Histological subtypes |  |  |  |
| Serous | N/A | 8369 (57.6) |  |
| Endometroid |  | 2067 (14.2) |  |
| Clear Cell |  | 1024 ( 7.1) |  |
| Mucinous |  | 943 ( 6.5) |  |
| Others^3^ |  | 2122 (14.6) |  |
|  |  |  |  |
| Endometriosis |  |  |  |
| No | 10030 (93.2) | 6647 (90.6) | <.0001 |
| Yes | 734 ( 6.8) | 689 ( 9.4) |  |
| age at menarche |  |  |  |
| ≤12 years old | 6730 (41.6) | 4113 (42.6) | 0.0995 |
| >12 | 9469 (58.4) | 5544 (57.4) |  |
| Number of full-term births |  |  |  |
| Mean± SD | 2.1± 1.5 | 1.8± 1.4 | <.0001 |
| No: full-term births  Yes: 1+ full-term births | 2263 (18.0)  10307 (82.0) | 2127 (23.0)  7117 (77.0) | <.0001 |

^1^ ovarian cancer in first degree relatives. ^2^ based on t-test for continuous variables and chi-square test for categorical variables. ^3^ includes mixed cell, other specified epithelial, undifferentiated, unknown (but known to be epithelial), nonepithelial, other or unknown if epithelial, or missing histology information.
