# Supplementary material for: Common Genetic Variation In Cellular Transport Genes and Epithelial Ovarian Cancer (EOC) Risk
Source: PLoS One. 2015 Jun 19;10(6):e0128106. doi: 10.1371/journal.pone.0128106 (PMC4474865; doi:10.1371/journal.pone.0128106)
Supplement: S2 Table — Significant associations are bolded. (DOCX) [file pone.0128106.s002.docx]

| **SNP** | **Gene** | **p_inv** | **OR_inv** | **p_ser** | **OR_ser** | **p_cc** | **OR_cc** | **p_end** | **OR_end** | **p_muc** | **OR_muc** | **p_LMP** | **OR_LMP** |
| --- | --- | --- | --- | --- | --- | --- | --- | --- | --- | --- | --- | --- | --- |
| rs5937960 | ABCB7 | 0.06081 | 1.71 (0.98-2.98) | **0.03846** | 1.93 (1.04-3.59) | 0.9103 | 0.89 (0.12-6.72) | 0.3379 | 1.71 (0.57-5.08) | 0.997 | 0 (0-.) | 0.172501358 | 2.45 (0.73-8.17) |
| rs7786151 | ABCF2 | 0.7503 | 1.01 (0.96-1.06) | 0.4658 | 1.02 (0.96-1.09) | 0.7189 | 0.97 (0.84-1.13) | 0.8743 | 1.01 (0.91-1.12) | 0.9865 | 1 (0.86-1.17) | **0.044553** | 1.13 (1-1.27) |
| rs10024579 | ACSL1 | **0.04644** | 1.03 (1-1.07) | 0.1055 | 1.03 (0.99-1.08) | 0.5364 | 0.97 (0.88-1.07) | 0.1482 | 1.05 (0.98-1.13) | 0.2165 | 1.07 (0.96-1.18) | 0.644900561 | 1.02 (0.94-1.1) |
| rs3822464 | AMACR | 0.2333 | 1.02 (0.99-1.05) | 0.5562 | 1.01 (0.97-1.05) | 0.8147 | 0.99 (0.9-1.09) | **0.03632** | 1.07 (1.01-1.15) | 0.327 | 1.05 (0.95-1.15) | 0.40462782 | 1.03 (0.96-1.11) |
| rs10941110 | AMACR | 0.3273 | 1.02 (0.98-1.05) | 0.628 | 1.01 (0.97-1.05) | 0.8779 | 1.01 (0.92-1.1) | **0.04473** | 1.07 (1-1.14) | 0.3168 | 1.05 (0.96-1.15) | 0.819610954 | 1 (0.94-1.08) |
| rs7215084 | ATP2A3 | **0.02802** | 1.03 (1-1.07) | 0.06518 | 1.04 (1-1.07) | **0.03655** | 1.1 (1.01-1.2) | **0.01529** | 1.08 (1.02-1.16) | 0.1662 | 1.07 (0.97-1.17) | 0.399323663 | 1.03 (0.96-1.11) |
| rs3745004 | ATP5A1 | 0.444 | 0.98 (0.94-1.03) | 0.6735 | 0.99 (0.93-1.05) | 0.4735 | 1.05 (0.92-1.21) | 0.1141 | 0.92 (0.83-1.02) | 0.637 | 1.04 (0.9-1.2) | **0.003432597** | 1.18 (1.06-1.31) |
| rs210359 | BMP4 | **0.04398** | 1.06 (1-1.13) | 0.07405 | 1.07 (0.99-1.15) | 0.7261 | 1.03 (0.86-1.23) | 0.9557 | 1 (0.87-1.14) | 0.3848 | 1.09 (0.9-1.31) | 0.786348324 | 0.98 (0.85-1.13) |
| rs573687 | CDKN2B | 0.2794 | 0.98 (0.95-1.01) | **0.04651** | 0.96 (0.93-1) | 0.4836 | 1.03 (0.94-1.13) | 0.4184 | 0.97 (0.91-1.04) | 0.793 | 0.99 (0.9-1.09) | 0.183818307 | 0.95 (0.98-1.02) |
| rs6736570 | CYP26B1 | **0.04551** | 1.03 (1-1.07) | 0.1793 | 1.03 (0.99-1.07) | 0.435 | 1.04 (0.94-1.14) | 0.2905 | 1.04 (0.97-1.11) | 0.7838 | 1.01 (0.92-1.12) | 0.740103154 | 0.99 (0.91-1.07) |
| rs10153788 | CYP26B1 | 0.2952 | 1.02 (0.99-1.05) | 0.3643 | 1.02 (0.98-1.06) | 0.861 | 0.99 (0.91-1.09) | 0.6465 | 1.02 (0.95-1.08) | 0.8107 | 0.99 (0.9-1.09) | **0.008819688** | 0.9 (0.85-0.98) |
| rs843354 | ECE2 | 0.4627 | 0.98 (0.92-1.04) | 0.8894 | 1.01 (0.93-1.08) | 0.2297 | 1.11 (0.94-1.32) | **0.002213** | 0.8 (0.7-0.92) | 0.1479 | 1.14 (0.96-1.35) | 0.590353852 | 0.96 (0.83-1.11) |
| rs4236723 | FUT10 | 0.4947 | 0.99 (0.96-1.02) | 0.6587 | 0.99 (0.96-1.03) | **0.03313** | 1.1 (1.01-1.21) | 0.1704 | 0.95 (0.89-1.02) | 0.6574 | 0.98 (0.89-1.08) | 0.982545217 | 0.99 (0.93-1.07) |
| rs16880128 | FUT10 | 0.5951 | 0.99 (0.96-1.02) | 0.9418 | 1 (0.96-1.04) | **0.01958** | 0.9 (0.82-0.98) | 0.9789 | 1 (0.94-1.07) | 0.8177 | 0.99 (0.9-1.09) | 0.900509618 | 0.99 (0.93-1.07) |
| rs4557662 | FUT10 | 0.821 | 1 (0.97-1.03) | 0.8321 | 1 (0.97-1.04) | **0.01631** | 0.89 (0.82-0.98) | 0.7542 | 1.01 (0.95-1.08) | 0.6065 | 0.98 (0.89-1.07) | 0.899695808 | 0.99 (0.93-1.07) |
| rs17216603 | HEPH | **0.000255** | 0.85 (0.77-0.93) | **0.0001994** | 0.81 (0.73-0.91) | 0.07007 | 0.77 (0.58-1.02) | 0.2596 | 0.9 (0.74-1.08) | 0.5565 | 0.92 (0.7-1.21) | **0.020629073** | 0.78 (0.63-0.97) |
| rs855211 | IGF1 | **0.03698** | 1.04 (1-1.09) | 0.1691 | 1.04 (0.99-1.09) | 0.4874 | 0.96 (0.85-1.08) | 0.2586 | 1.05 (0.96-1.15) | 0.4406 | 0.95 (0.84-1.08) | 0.551922122 | 0.97 (0.88-1.07) |
| rs2056576 | IL6 | 0.821 | 1 (0.96-1.03) | 0.771 | 1.01 (0.97-1.05) | 0.8463 | 0.99 (0.9-1.09) | 0.6657 | 0.98 (0.92-1.06) | 0.7237 | 0.98 (0.89-1.09) | **0.039437079** | 1.08 (1-1.17) |
| rs258434 | KLHDC5 | **0.03428** | 1.04 (1-1.07) | **0.01787** | 1.05 (1.01-1.1) | 0.4694 | 1.04 (0.94-1.15) | 0.2639 | 1.04 (0.97-1.12) | 0.6664 | 0.98 (0.88-1.09) | 0.141663128 | 1.06 (0.98-1.15) |
| rs956679 | KLHDC5 | **0.03713** | 1.04 (1-1.09) | 0.1115 | 1.04 (0.99-1.09) | 0.0503 | 1.12 (1-1.26) | 0.1078 | 1.07 (0.99-1.17) | 0.2241 | 1.08 (0.96-1.22) | 0.103703751 | 1.08 (0.98-1.19) |
| rs5973898 | LOC100131029 | 0.4323 | 1.19 (0.77-1.84) | 0.7801 | 0.92 (0.53-1.6) | 0.8661 | 0.88 (0.21-3.69) | 0.99 | 1.01 (0.39-2.58) | **0.008125** | 3.26 (1.36-7.84) | 0.332924186 | 1.6 (0.6-3.99) |
| rs5944317 | MAGEB18 | 0.5719 | 0.99 (0.96-1.02) | 0.585 | 0.99 (0.95-1.03) | 0.433 | 0.96 (0.88-1.06) | 0.7462 | 1.01 (0.95-1.08) | 0.9156 | 0.99 (0.91-1.09) | **0.048704758** | 1.07 (1.01-1.15) |
| rs4149186 | MGST1 | **0.04239** | 0.96 (0.93-1) | **0.03669** | 0.95 (0.91-1) | 0.3867 | 0.95 (0.85-1.06) | 0.8996 | 1 (0.92-1.08) | 0.4724 | 0.96 (0.86-1.07) | 0.881940614 | 1 (0.92-1.1) |
| rs6488840 | MGST1 | **0.04763** | 0.96 (0.93-1) | **0.042** | 0.95 (0.91-1) | 0.4084 | 0.96 (0.86-1.07) | 0.9445 | 1 (0.92-1.08) | 0.4801 | 0.96 (0.86-1.08) | 0.90242534 | 1 (0.92-1.09) |
| rs12849510 | MID2 | **0.03669** | 1.08 (1.01-1.16) | **0.006496** | 1.12 (1.03-1.22) | 0.7644 | 0.97 (0.78-1.2) | 0.1702 | 1.11 (0.96-1.29) | 0.8083 | 0.97 (0.77-1.23) | 0.75867633 | 1.03 (0.87-1.21) |
| rs1950902 | MTHFD1 | 0.8302 | 1 (0.96-1.04) | 0.3765 | 0.98 (0.93-1.03) | **0.02274** | 1.14 (1.02-1.27) | 0.7465 | 1.01 (0.93-1.1) | 0.3948 | 1.05 (0.94-1.18) | 0.602434302 | 0.98 (0.89-1.07) |
| rs4607073 | RARB | 0.3541 | 1.02 (0.98-1.05) | **0.02572** | 1.04 (1.01-1.08) | 0.5197 | 0.97 (0.89-1.06) | 0.9629 | 1 (0.94-1.07) | **0.03437** | 0.9 (0.82-0.99) | 0.763485568 | 0.99 (0.92-1.06) |
| rs2986208 | S100A7 | 0.5144 | 0.99 (0.95-1.02) | 0.5967 | 1.01 (0.97-1.06) | 0.3335 | 1.05 (0.95-1.17) | **0.0475** | 0.92 (0.85-1) | 0.3699 | 0.95 (0.85-1.06) | 0.214900838 | 0.95 (0.97-1.03) |
| rs1804495 | SERPINA7 | **0.04187** | 1.05 (1-1.1) | **0.04499** | 1.06 (1-1.12) | **0.004166** | 1.21 (1.06-1.39) | 0.2851 | 1.06 (0.96-1.17) | **0.04482** | 0.85 (0.73-1) | **0.016017336** | 1.14 (1.03-1.27) |
| rs8072340 | SLC13A5 | 0.1804 | 1.02 (0.99-1.06) | 0.2726 | 1.02 (0.98-1.06) | **0.03565** | 1.11 (1.01-1.21) | 0.1269 | 1.05 (0.99-1.13) | 0.8136 | 1.01 (0.92-1.12) | 0.660044123 | 1.02 (0.94-1.1) |
| rs3810755 | SLC25A43 | 0.7923 | 1 (0.97-1.03) | 0.6722 | 0.99 (0.96-1.03) | 0.7779 | 0.99 (0.9-1.08) | 0.8221 | 1.01 (0.94-1.08) | **0.0459** | 1.1 (1-1.21) | 0.461991691 | 0.97 (0.9-1.05) |
| rs681309 | SLC25A45 | **0.02941** | 0.97 (0.94-1) | 0.9929 | 1 (0.96-1.04) | 0.5932 | 0.98 (0.89-1.07) | **0.003544** | 0.91 (0.85-0.97) | **0.012** | 0.89 (0.81-0.97) | 0.273533641 | 1.04 (0.97-1.12) |
| rs11140719 | SLC28A3 | 0.09223 | 0.94 (0.88-1.01) | 0.2572 | 0.95 (0.88-1.04) | 0.3776 | 0.91 (0.74-1.12) | **0.02806** | 0.84 (0.72-0.98) | 0.3178 | 1.11 (0.9-1.36) | 0.190638023 | 0.9 (0.76-1.06) |
| rs1439054 | SLC28A3 | 0.3755 | 0.98 (0.93-1.03) | 0.931 | 1 (0.94-1.06) | 0.3722 | 1.07 (0.92-1.25) | **0.007545** | 0.85 (0.75-0.96) | 0.8109 | 1.02 (0.87-1.2) | 0.576603405 | 0.97 (0.85-1.09) |
| rs11140694 | SLC28A3 | 0.6306 | 0.99 (0.95-1.03) | 0.9875 | 1 (0.95-1.05) | 0.2351 | 1.08 (0.95-1.22) | **0.01831** | 0.89 (0.81-0.98) | 0.8596 | 1.01 (0.88-1.16) | 0.309593299 | 0.95 (0.85-1.05) |
| rs5412 | SLC2A4 | **0.0188** | 1.05 (1.01-1.09) | 0.08188 | 1.04 (0.99-1.1) | **0.02605** | 1.14 (1.02-1.28) | 0.3359 | 1.04 (0.96-1.14) | 0.2235 | 1.08 (0.96-1.22) | 0.685081249 | 1.02 (0.93-1.12) |
| rs2654189 | SLC2A4 | 0.5925 | 0.99 (0.96-1.02) | 0.4246 | 1.02 (0.98-1.06) | **0.009949** | 0.88 (0.8-0.97) | 0.619 | 0.98 (0.92-1.05) | 0.6889 | 0.98 (0.89-1.08) | 0.689596981 | 0.98 (0.91-1.06) |
| rs9803645 | SLC2A7 | 0.1555 | 1.06 (0.98-1.14) | **0.00878** | 1.13 (1.03-1.24) | 0.7947 | 1.03 (0.82-1.3) | 0.6719 | 0.96 (0.81-1.14) | 0.1502 | 0.83 (0.64-1.07) | 0.506832039 | 1.06 (0.89-1.3) |
| rs12727395 | SLC30A1 | **0.002662** | 0.94 (0.91-0.98) | 0.2251 | 0.97 (0.93-1.02) | **0.001876** | 0.83 (0.74-0.94) | 0.1495 | 0.94 (0.87-1.02) | 0.09946 | 0.91 (0.81-1.02) | 0.386439824 | 0.96 (0.88-1.05) |
| rs338685 | SLC30A5 | 0.2711 | 1.02 (0.99-1.05) | 0.1577 | 1.03 (0.99-1.07) | 0.6398 | 0.98 (0.88-1.08) | 0.9986 | 1 (0.93-1.07) | 0.919 | 1.01 (0.91-1.12) | **0.023938951** | 0.9 (0.84-0.99) |
| rs212755 | SLC30A6 | 0.1409 | 1.02 (0.99-1.06) | 0.09363 | 1.03 (0.99-1.07) | 0.3858 | 1.04 (0.95-1.14) | 0.328 | 0.97 (0.91-1.03) | 0.4725 | 1.04 (0.94-1.14) | **0.031947722** | 1.08 (1.01-1.16) |
| rs6716179 | SLC30A6 | 0.1627 | 1.02 (0.99-1.05) | 0.09146 | 1.03 (0.99-1.07) | 0.4311 | 1.04 (0.95-1.13) | 0.2932 | 0.97 (0.91-1.03) | 0.5836 | 1.03 (0.94-1.13) | **0.020939628** | 1.09 (1.01-1.17) |
| rs212699 | SLC30A6 | 0.1884 | 1.02 (0.99-1.05) | 0.1877 | 1.03 (0.99-1.07) | 0.2792 | 1.05 (0.96-1.15) | 0.5611 | 0.98 (0.92-1.05) | 0.09241 | 1.09 (0.99-1.19) | **0.035368208** | 1.08 (1-1.16) |
| rs212745 | SLC30A6 | 0.2077 | 1.02 (0.99-1.05) | 0.1381 | 1.03 (0.99-1.07) | 0.2042 | 1.06 (0.97-1.16) | 0.32 | 0.97 (0.91-1.03) | 0.1714 | 1.07 (0.97-1.17) | **0.025160581** | 1.09 (1.01-1.17) |
| rs212758 | SLC30A6 | 0.2205 | 1.02 (0.99-1.05) | 0.2754 | 1.02 (0.98-1.06) | 0.4505 | 1.04 (0.94-1.14) | 0.3218 | 0.97 (0.9-1.03) | 0.1013 | 1.08 (0.98-1.19) | **0.011573382** | 1.1 (1.02-1.19) |
| rs13427170 | SLC30A6 | 0.2921 | 0.98 (0.95-1.01) | 0.1297 | 0.97 (0.94-1.01) | 0.4635 | 0.97 (0.89-1.06) | 0.2267 | 1.04 (0.98-1.11) | 0.9407 | 1 (0.91-1.09) | **0.010982603** | 0.91 (0.85-0.98) |
| rs10282940 | SLC30A8 | **0.01077** | 0.93 (0.89-0.98) | 0.5823 | 0.98 (0.92-1.05) | 0.1932 | 0.9 (0.77-1.05) | **0.03727** | 0.89 (0.79-0.99) | 0.2097 | 0.9 (0.76-1.06) | 0.369818876 | 0.96 (0.87-1.05) |
| rs10910292 | SLC35F3 | **0.02497** | 0.92 (0.85-0.99) | 0.07761 | 0.92 (0.84-1.01) | 0.705 | 0.96 (0.77-1.2) | 0.6612 | 1.04 (0.89-1.21) | 0.2428 | 0.87 (0.68-1.1) | 0.951715046 | 1 (0.84-1.2) |
| rs936411 | SLC35F3 | **0.04038** | 1.03 (1-1.06) | 0.3811 | 1.02 (0.98-1.05) | 0.7117 | 1.02 (0.93-1.11) | 0.05394 | 1.07 (1-1.14) | 0.08613 | 1.09 (0.99-1.19) | **0.017842881** | 1.09 (1.02-1.17) |
| rs10910353 | SLC35F3 | **0.04315** | 1.03 (1-1.06) | 0.3859 | 1.02 (0.98-1.05) | 0.665 | 1.02 (0.93-1.12) | 0.06781 | 1.06 (1-1.13) | 0.07626 | 1.09 (0.99-1.19) | **0.018485016** | 1.09 (1.014-1.18) |
| rs10910358 | SLC35F3 | **0.04561** | 1.03 (1-1.06) | 0.3289 | 1.02 (0.98-1.06) | 0.8006 | 1.01 (0.93-1.11) | 0.1167 | 1.05 (0.99-1.12) | 0.08912 | 1.08 (0.99-1.19) | **0.018759359** | 1.09 (1.10-1.17) |
| rs10910360 | SLC35F3 | **0.04808** | 1.03 (1-1.06) | 0.3477 | 1.02 (0.98-1.06) | 0.9152 | 1.01 (0.92-1.1) | 0.09851 | 1.06 (0.99-1.13) | 0.1284 | 1.08 (0.98-1.18) | **0.02900865** | 1.08 (1-1.16) |
| rs4339928 | SLC35F3 | 0.1075 | 1.03 (0.99-1.06) | 0.1687 | 1.03 (0.99-1.07) | 0.7277 | 1.02 (0.93-1.12) | **0.002502** | 1.11 (1.04-1.19) | 0.2422 | 0.94 (0.86-1.04) | 0.965743642 | 0.99 (0.93-1.08) |
| rs9435500 | SLC35F3 | 0.5411 | 1.01 (0.98-1.04) | 0.7481 | 1.01 (0.97-1.05) | **0.02204** | 1.12 (1.02-1.22) | 0.7221 | 0.99 (0.92-1.06) | 0.5956 | 0.97 (0.88-1.08) | 0.638671382 | 1.02 (0.94-1.1) |
| rs12141026 | SLC35F3 | 0.6441 | 1.01 (0.97-1.04) | 0.2816 | 1.02 (0.98-1.07) | 0.08253 | 1.09 (0.99-1.21) | 0.8357 | 1.01 (0.94-1.08) | 0.3585 | 0.95 (0.86-1.06) | **0.000870476** | 1.15 (1.06-1.24) |
| rs12089698 | SLC35F3 | 0.7093 | 1.01 (0.97-1.04) | 0.2863 | 1.02 (0.98-1.07) | 0.09329 | 1.09 (0.99-1.2) | 0.9279 | 1 (0.93-1.08) | 0.3164 | 0.95 (0.85-1.05) | **0.00093145** | 1.15 (1.06-1.23) |
| rs1552846 | SLC39A11 | **0.002631** | 0.95 (0.91-0.98) | **0.01452** | 0.95 (0.91-0.99) | 0.3401 | 0.95 (0.85-1.06) | **0.04645** | 0.92 (0.86-1) | 0.3646 | 0.95 (0.85-1.06) | 0.924331593 | 1 (0.92-1.09) |
| rs9908917 | SLC39A11 | **0.01437** | 0.95 (0.92-0.99) | **0.01012** | 0.94 (0.9-0.99) | 0.8195 | 1.01 (0.91-1.13) | 0.1615 | 0.94 (0.87-1.02) | 0.7037 | 0.98 (0.87-1.1) | **0.000392198** | 0.85 (0.77-0.93) |
| rs1177982 | SLC39A11 | 0.0687 | 0.97 (0.93-1) | **0.01329** | 0.95 (0.91-0.99) | 0.3836 | 1.05 (0.94-1.16) | 0.2322 | 0.95 (0.88-1.03) | 0.8443 | 1.01 (0.91-1.13) | 0.702154761 | 1.02 (0.94-1.01) |
| rs9899923 | SLC39A11 | 0.5057 | 0.99 (0.96-1.02) | 0.7844 | 1.01 (0.97-1.04) | 0.1538 | 0.94 (0.86-1.03) | 0.1865 | 0.96 (0.9-1.02) | 0.3142 | 0.95 (0.87-1.05) | **0.013550586** | 1.1 (1.02-1.18) |
| rs9905659 | SLC39A11 | 0.5644 | 1.01 (0.97-1.05) | 0.2742 | 1.03 (0.98-1.08) | **0.0445** | 0.88 (0.79-1) | 0.608 | 0.98 (0.9-1.06) | 0.8266 | 1.01 (0.9-1.14) | 0.292617324 | 1.05 (0.96-1.15) |
| rs9913553 | SLC39A11 | 0.7408 | 0.99 (0.94-1.04) | 0.5361 | 0.98 (0.92-1.04) | 0.7633 | 0.98 (0.84-1.13) | 0.3365 | 0.95 (0.85-1.06) | **0.0475** | 1.16 (1-1.34) | 0.471263866 | 1.04 (0.93-1.17) |
| rs8068946 | SLC39A11 | 0.9065 | 1 (0.97-1.03) | 0.3731 | 1.02 (0.98-1.06) | **0.008281** | 0.88 (0.8-0.97) | 0.4833 | 0.98 (0.91-1.05) | 0.7346 | 0.98 (0.89-1.09) | 0.460439529 | 1.03 (0.95-1.11) |
| rs151399 | SLC39A8 | 0.1885 | 0.98 (0.94-1.01) | 0.3372 | 0.98 (0.94-1.02) | 0.1782 | 1.08 (0.97-1.2) | **0.04612** | 0.92 (0.85-1) | 0.2599 | 1.07 (0.95-1.19) | 0.538755057 | 1.03 (0.94-1.12) |
| rs10049581 | SLC39A8 | 0.4054 | 0.98 (0.93-1.03) | 0.5648 | 0.98 (0.92-1.05) | **0.03313** | 0.84 (0.71-0.99) | 0.9099 | 1.01 (0.9-1.12) | 0.7903 | 1.02 (0.87-1.19) | 0.698043691 | 0.98 (0.86-1.1) |
| rs2997380 | SLC44A3 | **0.03464** | 1.03 (1-1.07) | 0.1297 | 1.03 (0.99-1.07) | 0.3167 | 1.05 (0.96-1.15) | 0.2063 | 1.04 (0.98-1.11) | 0.9202 | 1.01 (0.91-1.11) | **0.047356982** | 1.08 (1.001-1.16) |
| rs3933012 | SLC44A3 | **0.04339** | 1.03 (1-1.07) | 0.2656 | 1.02 (0.98-1.06) | 0.2441 | 1.06 (0.96-1.16) | 0.1364 | 1.05 (0.98-1.13) | 0.2863 | 1.06 (0.96-1.16) | **0.024338329** | 1.09 (1.01-1.17) |
| rs2933912 | SLC44A3 | 0.8509 | 1 (0.97-1.04) | 0.71 | 0.99 (0.96-1.03) | 0.2634 | 0.95 (0.86-1.04) | **0.01624** | 1.09 (1.02-1.16) | 0.9634 | 1 (0.91-1.1) | 0.985508041 | 1 (0.93-1.08) |
| rs10493559 | SLC44A5 | 0.1224 | 1.06 (0.98-1.14) | 0.967 | 1 (0.91-1.09) | 0.189 | 1.15 (0.93-1.42) | **0.02648** | 1.18 (1.02-1.37) | 0.1893 | 1.15 (0.93-1.43) | 0.082288107 | 1.16 (0.98-1.37) |
| rs4839225 | SLC6A17 | 0.4442 | 0.99 (0.96-1.02) | 0.9686 | 1 (0.96-1.04) | 0.9052 | 0.99 (0.91-1.09) | **0.01273** | 0.92 (0.86-0.98) | 0.9197 | 1.01 (0.91-1.11) | 0.098294489 | 1.07 (0.99-1.15) |
| rs4839223 | SLC6A17 | 0.4853 | 0.99 (0.96-1.02) | 0.9793 | 1 (0.96-1.04) | 0.911 | 0.99 (0.91-1.09) | **0.01624** | 0.92 (0.86-0.98) | 0.9503 | 1 (0.91-1.11) | 0.081778329 | 1.07 (0.99-1.15) |
| rs16831558 | SLC6A9 | 0.7367 | 1.01 (0.96-1.06) | 0.5382 | 1.02 (0.96-1.08) | 0.1732 | 0.91 (0.79-1.04) | 0.6348 | 1.02 (0.93-1.13) | 0.3862 | 1.06 (0.93-1.22) | **0.039246171** | 1.17 (1-1.24) |
| rs6525447 | SLC7A3 | 0.6847 | 1.01 (0.96-1.07) | 0.7445 | 0.99 (0.93-1.06) | 0.06359 | 1.15 (0.99-1.34) | 0.1649 | 1.08 (0.97-1.21) | **0.03085** | 0.82 (0.69-0.98) | 0.334630987 | 1.06 (0.94-1.2) |
| rs11247611 | SLC9A1 | 0.9552 | 1 (0.95-1.05) | 0.5944 | 1.02 (0.96-1.08) | 0.9871 | 1 (0.87-1.15) | 0.9049 | 1.01 (0.91-1.11) | **0.04378** | 0.85 (0.73-1) | 0.517158175 | 0.96 (0.86-1.08) |
| rs7751646 | TNFRSF21 | **0.003961** | 1.05 (1.01-1.08) | **0.03251** | 1.04 (1-1.08) | 0.2723 | 1.05 (0.96-1.15) | 0.2498 | 1.04 (0.97-1.11) | 0.1299 | 1.08 (0.98-1.18) | 0.398302522 | 1.03 (0.96-1.11) |
| rs6608821 | TSPAN7 | 0.7795 | 0.99 (0.95-1.04) | 0.9236 | 1 (0.94-1.06) | 0.5245 | 0.95 (0.83-1.1) | 0.2001 | 1.07 (0.97-1.18) | 0.3436 | 0.93 (0.8-1.08) | **0.047741852** | 1.12 (1.00-1.25) |
| rs11563251 | UGT1A5 | 0.05018 | 0.95 (0.91-1) | 0.07415 | 0.95 (0.89-1.01) | 0.7301 | 1.03 (0.89-1.18) | **0.0006803** | 0.82 (0.74-0.92) | 0.486 | 1.06 (0.91-1.22) | 0.210535524 | 1.07 (0.96-1.2) |
| rs2703862 | UNC5D | 0.6563 | 1.01 (0.96-1.07) | 0.6385 | 0.99 (0.93-1.05) | 0.2916 | 1.08 (0.93-1.25) | 0.8071 | 1.01 (0.91-1.13) | **0.04724** | 1.16 (1-1.35) | **0.028009542** | 0.87 (0.77-0.99) |
| rs4741746 | VLDLR | **0.005082** | 0.94 (0.9-0.98) | **0.04328** | 0.95 (0.9-1) | 0.5031 | 0.96 (0.84-1.09) | 0.06299 | 0.91 (0.83-1.01) | 0.2755 | 0.92 (0.8-1.06) | 0.465827183 | 0.96 (0.85-1.08) |
| rs10967188 | VLDLR | **0.009741** | 0.95 (0.91-0.99) | 0.08109 | 0.96 (0.91-1.01) | 0.4711 | 0.96 (0.85-1.08) | **0.0407** | 0.91 (0.83-1) | 0.2933 | 0.93 (0.82-1.06) | 0.262472295 | 0.94 (0.85-1.05) |
| rs734312 | WFS1 | 0.1461 | 1.02 (0.99-1.06) | **0.02427** | 1.04 (1.01-1.08) | 0.7682 | 0.99 (0.9-1.08) | 0.4689 | 1.02 (0.96-1.09) | 0.3426 | 0.96 (0.87-1.05) | 0.42491158 | 0.97 (0.9-1.04) |
