# Supplementary material for: Common Genetic Variation In Cellular Transport Genes and Epithelial Ovarian Cancer (EOC) Risk
Source: PLoS One. 2015 Jun 19;10(6):e0128106. doi: 10.1371/journal.pone.0128106 (PMC4474865; doi:10.1371/journal.pone.0128106)
Supplement: S3 Table — SNPs are sorted by p-values. (DOCX) [file pone.0128106.s003.docx]

| **SNP** | **p-value** | **OR (95% CI)** | **Gene** |
| --- | --- | --- | --- |
| rs117729793 | 0.000566 | 2.55 (1.5-4.35) | SLC39A11 |
| rs143731556 | 0.0009 | 0.93 (0.89-0.97) | SLC39A11 |
| rs113471799 | 0.00125 | 0.93 (0.88-0.97) | SLC39A11 |
| rs70896279 | 0.00144 | 1.05 (1.02-1.08) | SLC39A11 |
| rs139411155 | 0.00147 | 0.66 (0.5-0.85) | SLC39A11 |
| rs28463307 | 0.00156 | 0.91 (0.86-0.97) | SLC39A11 |
| rs67914355 | 0.00175 | 0.95 (0.92-0.98) | SLC39A11 |
| rs11601539 | 0.00179 | 1.12 (1.04-1.2) | SLC25A45 |
| rs12600622 | 0.00188 | 0.93 (0.89-0.97) | SLC39A11 |
| rs4479318 | 0.00192 | 0.94 (0.91-0.98) | SLC39A11 |
| rs17863762 | 0.00198 | 0.84 (0.76-0.94) | UGT1A |
| rs75528157 | 0.00199 | 0.93 (0.89-0.97) | SLC39A11 |
| rs8065256 | 0.00199 | 0.93 (0.89-0.97) | SLC39A11 |
| rs191937687 | 0.00214 | 0.3 (0.14-0.64) | SLC39A11 |
| rs67913395 | 0.00255 | 0.95 (0.92-0.98) | SLC39A11 |
| rs11077657 | 0.00269 | 1.06 (1.02-1.1) | SLC39A11 |
| rs139049504 | 0.00304 | 1.13 (1.04-1.22) | SLC25A45 |
| rs117909784 | 0.00316 | 1.48 (1.14-1.92) | SLC39A11 |
| rs17216582 | 0.00339 | 0.87 (0.8-0.96) | HEPH |
| rs66715441 | 0.00355 | 0.95 (0.92-0.98) | SLC39A11 |
| rs117877946 | 0.00397 | 1.34 (1.1-1.65) | SLC39A11 |
| rs66479497 | 0.00408 | 0.95 (0.92-0.98) | SLC39A11 |
| rs141170367 | 0.00425 | 1.41 (1.11-1.79) | SLC39A11 |
| rs72937117 | 0.00453 | 0.87 (0.79-0.96) | SLC25A45 |
| rs8082220 | 0.00459 | 1.05 (1.01-1.08) | SLC39A11 |
| rs67834372 | 0.00463 | 0.95 (0.92-0.99) | SLC39A11 |
| rs70899090 | 0.00474 | 1.04 (1.01-1.07) | SLC39A11 |
| rs72847915 | 0.00476 | 0.93 (0.88-0.98) | SLC39A11 |
| rs78863042 | 0.0048 | 0.96 (0.93-0.99) | SLC39A11 |
| rs654312 | 0.00487 | 0.88 (0.81-0.96) | HEPH |
| rs62613170 | 0.00488 | 0.88 (0.81-0.96) | HEPH |
| rs116312632 | 0.00504 | 0.89 (0.82-0.96) | SLC25A45 |
| rs12452069 | 0.00505 | 1.04 (1.01-1.07) | SLC39A11 |
| rs116958972 | 0.00516 | 1.86 (1.2-2.88) | UGT1A |
| rs8075452 | 0.0052 | 0.95 (0.91-0.98) | SLC39A11 |
| rs4246434 | 0.00526 | 1.06 (1.02-1.1) | SLC39A11 |
| rs12051814 | 0.00541 | 1.04 (1.01-1.07) | SLC39A11 |
| rs17868329 | 0.00553 | 0.8 (0.68-0.93) | UGT1A |
| rs4969125 | 0.00555 | 1.04 (1.01-1.07) | SLC39A11 |
| rs70896234 | 0.00557 | 1.04 (1.01-1.07) | SLC39A11 |
| rs72850948 | 0.00575 | 1.15 (1.04-1.27) | SLC39A11 |
| rs144745173 | 0.00597 | 0.51 (0.31-0.82) | SLC25A45 |
| rs7215293 | 0.00597 | 1.04 (1.01-1.07) | SLC39A11 |
| rs4598977 | 0.00602 | 1.04 (1.01-1.07) | SLC39A11 |
| rs12051560 | 0.00609 | 1.04 (1.01-1.07) | SLC39A11 |
| rs4246435 | 0.0062 | 1.05 (1.02-1.1) | SLC39A11 |
| rs55917507 | 0.0063 | 0.9 (0.83-0.97) | UGT1A |
| rs28507798 | 0.00656 | 1.05 (1.01-1.08) | SLC39A11 |
| rs7502714 | 0.00659 | 1.04 (1.01-1.07) | SLC39A11 |
| rs4969033 | 0.00662 | 1.04 (1.01-1.07) | SLC39A11 |
| rs7209250 | 0.00672 | 0.94 (0.9-0.98) | SLC39A11 |
| rs185990675 | 0.00683 | 1.67 (1.15-2.43) | SLC39A11 |
| rs7502006 | 0.00685 | 1.04 (1.01-1.07) | SLC39A11 |
| rs12051813 | 0.00691 | 1.04 (1.01-1.07) | SLC39A11 |
| rs7405650 | 0.00697 | 1.04 (1.01-1.07) | SLC39A11 |
| rs145442606 | 0.00705 | 1.37 (1.09-1.72) | SLC39A11 |
| rs149437502 | 0.00714 | 1.93 (1.2-3.12) | MGST1 |
| rs4614795 | 0.00725 | 1.04 (1.01-1.07) | SLC39A11 |
| rs80328419 | 0.00729 | 1.1 (1.03-1.18) | SLC25A45 |
| rs17216603 | 0.00751 | 0.89 (0.81-0.97) | HEPH |
| rs2344990 | 0.00752 | 1.06 (1.01-1.1) | SLC39A11 |
| rs180962000 | 0.0077 | 1.62 (1.14-2.31) | SLC39A11 |
| rs9892344 | 0.00798 | 1.04 (1.01-1.07) | SLC39A11 |
| rs7215166 | 0.00818 | 1.04 (1.01-1.07) | SLC39A11 |
| rs187322339 | 0.00819 | 2.29 (1.24-4.24) | SLC39A11 |
| rs1552846 | 0.00823 | 1.05 (1.01-1.08) | SLC39A11 |
| rs12453980 | 0.00828 | 1.04 (1.01-1.07) | SLC39A11 |
| rs35857574 | 0.00836 | 0.96 (0.93-0.99) | SLC39A11 |
| rs189013989 | 0.00876 | 1.98 (1.19-3.32) | SLC25A45 |
| rs67181829 | 0.0089 | 0.96 (0.93-0.99) | SLC39A11 |
| rs70895176 | 0.0092 | 1.04 (1.01-1.07) | SLC39A11 |
| rs35337077 | 0.0092 | 0.96 (0.93-0.99) | SLC39A11 |
| rs7406887 | 0.00926 | 1.04 (1.01-1.07) | SLC39A11 |
| rs79744439 | 0.00932 | 0.96 (0.93-0.99) | SLC39A11 |
| rs65143701 | 0.00933 | 1.04 (1.01-1.07) | SLC25A45 |
| rs4563093 | 0.00937 | 1.04 (1.01-1.07) | SLC39A11 |
| rs4969132 | 0.0101 | 1.04 (1.01-1.07) | SLC39A11 |
| rs12051632 | 0.0101 | 1.04 (1.01-1.07) | SLC39A11 |
| rs7217743 | 0.0101 | 1.04 (1.01-1.07) | SLC39A11 |
| rs7219995 | 0.0101 | 1.04 (1.01-1.07) | SLC39A11 |
| rs4969131 | 0.0102 | 1.04 (1.01-1.07) | SLC39A11 |
| rs28366752 | 0.0107 | 1.04 (1.01-1.08) | SLC39A11 |
| rs66661510 | 0.0109 | 0.96 (0.93-0.99) | SLC39A11 |
| rs70906911 | 0.011 | 0.96 (0.93-0.99) | SLC39A11 |
| rs585210 | 0.0111 | 1.04 (1.01-1.07) | SLC25A45 |
| rs11654304 | 0.0113 | 1.04 (1.01-1.07) | SLC39A11 |
| rs9895836 | 0.0114 | 1.04 (1.01-1.07) | SLC39A11 |
| rs62073071 | 0.0115 | 0.95 (0.91-0.99) | SLC39A11 |
| rs74420932 | 0.012 | 1.13 (1.03-1.24) | SLC39A11 |
| rs70897100 | 0.0121 | 1.04 (1.01-1.07) | SLC39A11 |
| rs3924957 | 0.0127 | 0.93 (0.88-0.98) | SLC39A11 |
| rs34675354 | 0.0127 | 0.95 (0.91-0.99) | SLC39A11 |
| rs4149196 | 0.0128 | 1.53 (1.1-2.12) | MGST1 |
| rs67180784 | 0.0128 | 0.96 (0.93-0.99) | SLC39A11 |
| rs8077363 | 0.0135 | 1.04 (1.01-1.07) | SLC39A11 |
| rs652501 | 0.0136 | 1.04 (1.01-1.07) | SLC25A45 |
| rs8077955 | 0.0136 | 1.04 (1.01-1.07) | SLC39A11 |
| rs113233842 | 0.0139 | 2.11 (1.16-3.83) | SLC39A11 |
| rs2073800 | 0.014 | 1.04 (1.01-1.07) | SLC25A45 |
| rs9908917 | 0.014 | 0.96 (0.92-0.99) | SLC39A11 |
| rs547221 | 0.0141 | 1.04 (1.01-1.07) | SLC25A45 |
| rs3924958 | 0.0141 | 0.93 (0.88-0.99) | SLC39A11 |
| rs70893210 | 0.0143 | 1.04 (1.01-1.07) | SLC39A11 |
| rs140385271 | 0.0144 | 2.31 (1.18-4.51) | SLC39A11 |
| rs4930154 | 0.0146 | 0.96 (0.94-0.99) | SLC25A45 |
| rs8066482 | 0.0148 | 1.04 (1.01-1.07) | SLC39A11 |
| rs72850972 | 0.0148 | 1.05 (1.01-1.09) | SLC39A11 |
| rs181484321 | 0.0151 | 1.55 (1.09-2.21) | SLC39A11 |
| rs7940559 | 0.0154 | 0.96 (0.93-0.99) | SLC25A45 |
| rs578270 | 0.0156 | 1.04 (1.01-1.07) | SLC25A45 |
| rs72843296 | 0.0156 | 0.89 (0.82-0.98) | SLC39A11 |
| rs73350937 | 0.0156 | 1.11 (1.02-1.21) | SLC39A11 |
| rs11551353 | 0.0158 | 1.09 (1.02-1.17) | SLC25A45 |
| rs71380144 | 0.0159 | 0.96 (0.93-0.99) | SLC39A11 |
| rs28898617 | 0.016 | 1.6 (1.09-2.34) | UGT1A |
| rs180820014 | 0.0165 | 1.7 (1.1-2.61) | MGST1 |
| rs111769016 | 0.0165 | 0.7 (0.52-0.94) | SLC39A11 |
| rs59067019 | 0.0166 | 1.11 (1.02-1.2) | SLC39A11 |
| rs681309 | 0.0167 | 1.04 (1.01-1.07) | SLC25A45 |
| rs190857327 | 0.0167 | 0.75 (0.6-0.95) | SLC39A11 |
| rs666194 | 0.0174 | 1.04 (1.01-1.07) | SLC25A45 |
| rs115488315 | 0.0175 | 0.37 (0.17-0.84) | SLC39A11 |
| rs57573748 | 0.0177 | 1.11 (1.02-1.2) | SLC39A11 |
| rs56964679 | 0.0177 | 1.11 (1.02-1.2) | SLC39A11 |
| rs61243278 | 0.0177 | 1.11 (1.02-1.2) | SLC39A11 |
| rs66528169 | 0.0181 | 0.96 (0.94-0.99) | SLC25A45 |
| rs70895174 | 0.0186 | 1.04 (1.01-1.07) | SLC39A11 |
| rs188383299 | 0.0187 | 0.63 (0.43-0.93) | SLC39A11 |
| rs2239675 | 0.0191 | 1.05 (1.01-1.1) | MGST1 |
| rs61736066 | 0.0201 | 0.93 (0.88-0.99) | SLC39A11 |
| rs70907554 | 0.0203 | 0.96 (0.93-0.99) | SLC39A11 |
| rs116656225 | 0.0207 | 1.45 (1.06-2) | SLC39A11 |
| rs36088096 | 0.0207 | 0.95 (0.9-0.99) | SLC39A11 |
| rs146788152 | 0.0208 | 2.1 (1.12-3.92) | SLC39A11 |
| rs11568318 | 0.021 | 0.93 (0.87-0.99) | UGT1A |
| rs9894801 | 0.0211 | 0.78 (0.64-0.96) | SLC39A11 |
| rs145020136 | 0.0212 | 1.75 (1.09-2.83) | SLC39A11 |
| rs117035129 | 0.0215 | 1.35 (1.05-1.74) | SLC39A11 |
| rs71032582 | 0.0217 | 0.95 (0.92-0.99) | SLC39A11 |
| rs182570656 | 0.0219 | 1.25 (1.03-1.51) | UGT1A |
| rs78272455 | 0.022 | 1.38 (1.05-1.81) | SLC39A11 |
| rs145738695 | 0.0222 | 0.66 (0.46-0.94) | SLC39A11 |
| rs140248949 | 0.0222 | 1.38 (1.05-1.81) | SLC39A11 |
| rs189517335 | 0.0226 | 0.86 (0.75-0.98) | SLC25A45 |
| rs190553425 | 0.0235 | 0.57 (0.35-0.93) | SLC25A45 |
| rs34916116 | 0.0245 | 0.93 (0.87-0.99) | UGT1A |
| rs17868341 | 0.025 | 0.93 (0.87-0.99) | UGT1A |
| rs17863800 | 0.0251 | 0.93 (0.88-0.99) | UGT1A |
| rs7608888 | 0.0256 | 0.91 (0.84-0.99) | UGT1A |
| rs115197260 | 0.0261 | 1.41 (1.04-1.9) | SLC39A11 |
| rs17874945 | 0.0264 | 0.93 (0.88-0.99) | UGT1A |
| rs62613167 | 0.027 | 0.91 (0.84-0.99) | HEPH |
| rs73350949 | 0.027 | 1.1 (1.01-1.2) | SLC39A11 |
| rs118085693 | 0.0274 | 0.83 (0.71-0.98) | SLC39A11 |
| rs192867208 | 0.0274 | 1.31 (1.03-1.66) | SLC39A11 |
| rs234661987 | 0.0279 | 0.93 (0.88-0.99) | UGT1A |
| rs74787288 | 0.0281 | 0.93 (0.88-0.99) | UGT1A |
| rs149136104 | 0.0283 | 1.26 (1.02-1.55) | SLC39A11 |
| rs71380142 | 0.0283 | 0.97 (0.94-1) | SLC39A11 |
| rs11888492 | 0.0283 | 0.95 (0.9-0.99) | UGT1A |
| rs75723458 | 0.0284 | 1.17 (1.02-1.36) | SLC25A45 |
| rs35799684 | 0.0284 | 0.97 (0.94-1) | SLC39A11 |
| rs111706856 | 0.0285 | 0.93 (0.88-0.99) | UGT1A |
| rs193230127 | 0.0286 | 1.58 (1.05-2.38) | SLC39A11 |
| rs111616126 | 0.0286 | 1.09 (1.01-1.17) | SLC39A11 |
| rs190245757 | 0.0287 | 1.31 (1.03-1.68) | SLC39A11 |
| rs234656069 | 0.029 | 0.93 (0.88-0.99) | UGT1A |
| rs72937115 | 0.0291 | 0.91 (0.84-0.99) | SLC25A45 |
| rs113250885 | 0.0296 | 0.93 (0.87-0.99) | UGT1A |
| rs70897129 | 0.0297 | 1.04 (1-1.07) | SLC39A11 |
| rs17862874 | 0.0297 | 0.93 (0.88-0.99) | UGT1A |
| rs17868338 | 0.0299 | 0.93 (0.88-0.99) | UGT1A |
| rs17863795 | 0.0301 | 0.93 (0.88-0.99) | UGT1A |
| rs183916174 | 0.0302 | 1.59 (1.05-2.42) | SLC39A11 |
| rs9894369 | 0.0304 | 1.03 (1-1.06) | SLC39A11 |
| rs188857965 | 0.0306 | 1.36 (1.03-1.8) | SLC39A11 |
| rs57630662 | 0.0307 | 0.87 (0.77-0.99) | SLC39A11 |
| rs45507691 | 0.031 | 0.93 (0.88-0.99) | UGT1A |
| rs79983684 | 0.0311 | 0.93 (0.88-0.99) | UGT1A |
| rs148715410 | 0.0313 | 0.38 (0.16-0.92) | SLC39A11 |
| rs141849530 | 0.0313 | 0.77 (0.61-0.98) | SLC39A11 |
| rs146321237 | 0.0316 | 0.53 (0.3-0.94) | UGT1A |
| rs67947981 | 0.0318 | 0.97 (0.94-1) | SLC39A11 |
| rs11655379 | 0.0322 | 1.05 (1-1.09) | SLC39A11 |
| rs72847997 | 0.0322 | 0.97 (0.94-1) | SLC39A11 |
| rs186439071 | 0.0324 | 0.87 (0.76-0.99) | SLC25A45 |
| rs17183190 | 0.0326 | 0.96 (0.92-1) | SLC39A11 |
| rs140271398 | 0.033 | 1.09 (1.01-1.17) | SLC39A11 |
| rs16977497 | 0.033 | 1.11 (1.01-1.21) | SLC39A11 |
| rs34925518 | 0.0332 | 0.96 (0.93-1) | SLC39A11 |
| rs74977278 | 0.0333 | 1.46 (1.03-2.07) | SLC39A11 |
| rs190133204 | 0.0334 | 1.56 (1.04-2.35) | SLC39A11 |
| rs183308239 | 0.0335 | 0.94 (0.88-0.99) | UGT1A |
| rs651283 | 0.0339 | 1.03 (1-1.06) | SLC25A45 |
| rs191203081 | 0.034 | 2.33 (1.06-5.09) | SLC39A11 |
| rs184500756 | 0.0344 | 0.79 (0.63-0.98) | SLC39A11 |
| rs70897131 | 0.0347 | 1.04 (1-1.07) | SLC39A11 |
| rs9915558 | 0.0348 | 1.04 (1-1.08) | SLC39A11 |
| rs148500488 | 0.0349 | 0.77 (0.6-0.98) | SLC39A11 |
| rs11563251 | 0.0353 | 0.95 (0.91-1) | UGT1A |
| rs146605355 | 0.0355 | 1.29 (1.02-1.64) | SLC39A11 |
| rs67608147 | 0.0357 | 0.96 (0.92-1) | SLC39A11 |
| rs79527388 | 0.036 | 1.1 (1.01-1.21) | SLC39A11 |
| rs70817099 | 0.0361 | 0.95 (0.9-1) | SLC39A11 |
| rs118010014 | 0.0361 | 1.1 (1.01-1.21) | SLC39A11 |
| rs11654812 | 0.0363 | 1.09 (1.01-1.17) | SLC39A11 |
| rs9332894 | 0.0364 | 1.16 (1.01-1.34) | MGST1 |
| rs141644129 | 0.0366 | 2.43 (1.06-5.59) | SLC39A11 |
| rs77600287 | 0.0366 | 0.94 (0.88-1) | UGT1A |
| rs65173481 | 0.0373 | 0.88 (0.77-0.99) | SLC25A45 |
| rs76642368 | 0.0376 | 1.09 (1-1.17) | SLC39A11 |
| rs6755571 | 0.0377 | 0.94 (0.88-1) | UGT1A |
| rs11651230 | 0.0378 | 1.05 (1-1.11) | SLC39A11 |
| rs184800720 | 0.0379 | 1.29 (1.01-1.64) | UGT1A |
| rs17868336 | 0.0381 | 0.92 (0.85-1) | UGT1A |
| rs28898590 | 0.0383 | 0.94 (0.88-1) | UGT1A |
| rs148502163 | 0.0387 | 0.88 (0.77-0.99) | SLC39A11 |
| rs17827723 | 0.039 | 1.34 (1.01-1.76) | SLC39A11 |
| rs17183634 | 0.0391 | 0.94 (0.88-1) | SLC39A11 |
| rs72848000 | 0.0393 | 1.08 (1-1.16) | SLC39A11 |
| rs77358763 | 0.0393 | 0.94 (0.88-1) | UGT1A |
| rs145916710 | 0.0396 | 1.32 (1.01-1.73) | SLC39A11 |
| rs9909293 | 0.0396 | 1.04 (1-1.08) | SLC39A11 |
| rs139986462 | 0.0397 | 0.67 (0.46-0.98) | SLC39A11 |
| rs16977498 | 0.0397 | 0.88 (0.79-0.99) | SLC39A11 |
| rs9901990 | 0.0401 | 0.97 (0.94-1) | SLC39A11 |
| rs70818875 | 0.0403 | 0.86 (0.74-0.99) | SLC39A11 |
| rs183286545 | 0.0405 | 1.39 (1.01-1.91) | SLC39A11 |
| rs11077634 | 0.0405 | 0.96 (0.92-1) | SLC39A11 |
| rs70908384 | 0.0407 | 0.97 (0.94-1) | SLC39A11 |
| rs1558764 | 0.041 | 1.03 (1-1.07) | MGST1 |
| rs2239676 | 0.0411 | 1.05 (1-1.11) | MGST1 |
| rs147373065 | 0.0412 | 0.6 (0.37-0.98) | SLC39A11 |
| rs185605986 | 0.0414 | 1.77 (1.02-3.06) | UGT1A |
| rs145852400 | 0.0421 | 1.15 (1-1.32) | SLC39A11 |
| rs149247216 | 0.0423 | 0.94 (0.88-1) | UGT1A |
| rs77916212 | 0.0425 | 1.08 (1-1.17) | SLC39A11 |
| rs80200357 | 0.0427 | 0.97 (0.94-1) | SLC39A11 |
| rs60653817 | 0.043 | 1.05 (1-1.09) | SLC39A11 |
| rs17864671 | 0.043 | 0.94 (0.88-1) | UGT1A |
| rs143190044 | 0.043 | 0.94 (0.88-1) | UGT1A |
| rs149455122 | 0.0432 | 1.2 (1.01-1.42) | SLC25A45 |
| rs35069052 | 0.0434 | 1.16 (1-1.33) | SLC39A11 |
| rs12949898 | 0.0434 | 0.95 (0.91-1) | SLC39A11 |
| rs6714486 | 0.0437 | 0.94 (0.88-1) | UGT1A |
| rs75420349 | 0.0442 | 0.89 (0.79-1) | SLC39A11 |
| rs34357742 | 0.0442 | 0.95 (0.91-1) | SLC39A11 |
| rs17862835 | 0.0442 | 0.94 (0.88-1) | UGT1A |
| rs192115968 | 0.0444 | 0.32 (0.1-0.97) | SLC39A11 |
| rs191059304 | 0.0446 | 1.65 (1.01-2.69) | SLC39A11 |
| rs184449990 | 0.0448 | 1.66 (1.01-2.71) | SLC39A11 |
| rs2452927 | 0.0452 | 0.79 (0.63-1) | SLC39A11 |
| rs78555352 | 0.0455 | 1.36 (1.01-1.83) | SLC39A11 |
| rs149952666 | 0.0458 | 0.69 (0.48-0.99) | SLC39A11 |
| rs144474364 | 0.0464 | 2.12 (1.01-4.45) | SLC39A11 |
| rs142949887 | 0.0464 | 3.1 (1.02-9.37) | SLC39A11 |
| rs35203651 | 0.0464 | 0.95 (0.91-1) | UGT1A |
| rs182470697 | 0.0469 | 0.46 (0.21-0.99) | UGT1A |
| rs117654830 | 0.0472 | 1.2 (1-1.43) | SLC39A11 |
| rs6501579 | 0.0476 | 1.03 (1-1.06) | SLC39A11 |
| rs192687379 | 0.0479 | 0.61 (0.38-1) | SLC39A11 |
| rs12941998 | 0.0479 | 0.95 (0.9-1) | SLC39A11 |
| rs71380136 | 0.0481 | 1.21 (1-1.47) | SLC39A11 |
| rs116727752 | 0.0481 | 0.94 (0.88-1) | UGT1A |
| rs139263463 | 0.0486 | 0.87 (0.77-1) | SLC39A11 |
| rs16519560 | 0.0491 | 1.03 (1-1.06) | MGST1 |
| rs77992942 | 0.0491 | 0.97 (0.94-1) | SLC39A11 |
| rs16977406 | 0.0494 | 1.21 (1-1.47) | SLC39A11 |
| rs143373661 | 0.0494 | 0.92 (0.84-1) | UGT1A |
| rs187935423 | 0.0496 | 0.55 (0.3-1) | SLC39A11 |
| rs146432524 | 0.0496 | 1.58 (1-2.48) | UGT1A |
| rs9332952 | 0.0497 | 1.03 (1-1.06) | MGST1 |
| rs143640842 | 0.0497 | 1.19 (1-1.42) | SLC39A11 |
| rs115642998 | 0.0498 | 0.94 (0.88-1) | UGT1A |
